# Patterns of contraceptive use among young Australian women with chronic disease: findings from a prospective cohort study

**DOI:** 10.1186/s12978-022-01413-x

**Published:** 2022-05-07

**Authors:** Melissa L. Harris, Nicholas Egan, Peta M. Forder, Deborah Bateson, Aaron L. Sverdlov, Vanessa E. Murphy, Deborah Loxton

**Affiliations:** 1 Centre for Women’s Health Research, College of Health, Medicine and Wellbeing, University of Newcastle, Newcastle, NSW Australia; 2 Hunter Medical Research Institute, Newcastle, NSW Australia; 3 Family Planning NSW, Ashfield, NSW Australia; 4 Discipline of Obstetrics, Gynaecology and Neonatology, Faculty of Medicine and Health, University of Sydney, Sydney, Australia; 5 School of Medicine and Public Health, University of Newcastle, Newcastle, NSW Australia; 6 Cardiovascular Department, John Hunter Hospital, Hunter New England Local Health District, Newcastle, NSW Australia; 7 Priority Research Centre for Healthy Lungs, College of Health, Medicine and Wellbeing, University of Newcastle, Newcastle, NSW Australia

**Keywords:** Chronic disease, Contraceptive methods, Long-acting reversible contraception, Pill, Withdrawal, Young women, Cohort study, Longitudinal

## Abstract

**Background:**

Given chronic disease is increasing among young women and unintended pregnancies among these women are associated with poor maternal and fetal outcomes, these women would benefit from effective preconception care. However, there is a lack of understanding of how these women use or don’t use contraception to inform such interventions. This study examined patterns of contraceptive use among an Australian cohort of young women and investigated the influence of chronic disease on contraceptive use over time.

**Methods:**

Using data from 15,244 young women from the Australian Longitudinal Study on Women’s Health (born 1989–1995), latent transition analysis was performed to identify distinct contraceptive patterns among women who were at risk of an unintended pregnancy. Multinomial mixed-effect models were used to evaluate the relationship between contraceptive combinations and chronic disease.

**Results:**

Contraceptive use for women with cardiac and autoinflammatory diseases differed to women without chronic disease over the observation period. Compared to women without chronic disease using the pill, women with cardiac disease had double the odds of using ‘other’ contraception and condoms (OR = 2.20, 95% CI 1.34, 3.59) and a modest increase in the odds of using the combined oral contraceptive pill and condoms (OR = 1.39, 95% CI 1.03, 1.89). Compared to women without chronic disease who used the pill, women with autoinflammatory disease had increased odds of using LARC and condoms (OR = 1.58, 95% CI 1.04, 2.41), using ‘other’ contraception and condoms (OR = 1.69, 95% CI 1.11, 2.57), and using the combined oral contraceptive pill and condoms (OR = 1.38, 95% CI 1.09, 1.75). No differences in contraceptive patterns over the observation period were found for women with asthma or diabetes when compared to women without chronic disease.

**Conclusion:**

The findings identified a need for effective contraceptive counselling as part of routine chronic disease care and improved communication between health care providers and women with chronic disease to improve young women’s contraceptive knowledge and agency in contraceptive choice, particularly for those with cardiac or autoinflammatory conditions. This may be the key to reducing high-risk unintended pregnancies among this vulnerable population.

**Supplementary Information:**

The online version contains supplementary material available at 10.1186/s12978-022-01413-x.

## Plain Language summary

Chronic disease is increasing among young women and unintended pregnancies among these women are associated with poor outcomes for both the mother and baby. To optimise outcomes, it is important for these women to plan pregnancies and use effective contraception until such time. However, there is a lack of understanding of how these women use or don’t use contraception, particularly with respect to highly effective contraception. This study examined patterns of contraceptive use among an Australian cohort of young women (born 1989–1995) and investigated the influence of chronic disease on contraceptive use over time. We found differences in contraceptive use over time for women with cardiac disease and those with autoinflammatory diseases. Importantly, compared to women without chronic disease using the pill alone, women with cardiac disease had double the odds of using low efficacy contraception. While women with autoinflammatory disease were 69% more likely to use long-acting methods combined with condoms, these women were also 70% more likely to use low efficacy contraception, compared to women without chronic disease who used the pill only. Contraceptive patterns did not differ for women with asthma or diabetes from women without chronic disease. The findings identified a need for effective contraceptive counselling as part of routine chronic disease care and improved communication between health care providers and women with chronic disease to improve young women’s contraceptive knowledge and contraceptive decision-making, particularly for those with cardiac or autoinflammatory conditions. This may be the key to reducing high-risk unintended pregnancies among this vulnerable population.

**Supplementary Information:**

The online version contains supplementary material available at 10.1186/s12978-022-01413-x.

## Background

Access to, and use of, effective contraception is the cornerstone of preconception care for women, allowing autonomous control over fertility and reproductive decisions. Although Australia has high access to contraception, unintended pregnancy remains an important public health issue, with around 40% of pregnancies unintended at conception [1]. All women of reproductive age may experience an unintended pregnancy, however certain sub-populations have been found to be at increased risk. There is mounting evidence to suggest that women with chronic disease experience unintended pregnancy at a higher rate than women without chronic disease, with rates in this population reported as high as 60% [2, 3]. For women with chronic disease, unintended pregnancies are associated with serious adverse maternal and perinatal outcomes, including congenital abnormalities, pre-term labour, spontaneous abortion, premature birth and fetal death [4]. Optimised preconception care and reproductive life planning is therefore critical to the prevention of unintended pregnancies and reduction in pregnancy-related complications for these women.

Despite this, there is a lack of high-quality evidence regarding how young women with chronic disease use, or don’t use, contraception, particularly in Australia. Of the few available international studies, the findings have been equivocal, in part due to their cross-sectional, retrospective nature and concentration on single disease entities with small samples [3, 5–7]. Cross-sectional studies fail to capture the dynamic nature of contraceptive use in different contexts over time, with contraceptive patterns found to vary according to a range of sociodemographic, lifestyle and sexual and reproductive health factors [8–11]. Additionally, none have specifically focused on women in early adulthood, the time of highest unintended pregnancy risk. In one of the only longitudinal studies available, only one-third of women with chronic disease (hypertension, asthma, hypothyroidism, diabetes, obesity, rheumatoid arthritis [RA], inflammatory bowel disease [IBD], or systemic lupus erythematosus [SLE]) [12] were found to be users of prescription contraception during the 3-year observation period compared to 41% of women without chronic disease. This study, however, was limited by a short time frame and reliance on insurance claims data. More recently, cross-sectional analysis of population-level data in the U.S. found substantially higher rates of contraceptive use among women of reproductive age with diabetes, cardiovascular disease, or asthma (87%) and when focused on contraceptive efficacy, contraceptive use differed by chronic disease type. Importantly, women with diabetes and cardiovascular disease were more likely to be users of less effective methods than women without chronic disease [13].

Chronic disease is on the rise among women of reproductive age in Australia (and increasing with successive generations) [14]. Further, contraceptive patterns differ markedly by age, with younger women reporting higher use of multiple (often less effective) methods than older women [15, 16]. This underscores the need to examine contraceptive patterns that better reflect ‘actual’ contraceptive practices. It is therefore imperative to use nationally representative population-level data to understand how contraceptive use changes over time among women with chronic disease to prevent unintended pregnancy in this high-risk population. This study examined patterns of contraceptive use among an Australian cohort of women born 1989–1995, and investigated the influence of chronic disease on contraceptive use over time.

## Methods

### Study design

Data were obtained from the 1989–1995 cohort of the Australian Longitudinal Study on Women’s Health (ALSWH), a national population-based study examining health and wellbeing among Australian women. Specific recruitment methods have been described in detailed elsewhere [17]. Briefly, women from this cohort were recruited through an open recruitment strategy involving a mix of online and offline methods including paid Facebook advertising, promotion using social and other internet-based media, paid and unpaid promotion through traditional media, and peer referral. Women were eligible for inclusion if they were aged 18–23 years in 2012–2013, had a Medicare number (Australia’s universal health insurance scheme) and consented to their survey data being linked to administrative health data. Women recruited through these methods were found to be demographically representative of similarly aged women in the Australian population, except for an overrepresentation of Australian-born and tertiary-educated women [18]. After the baseline survey in 2012–2013, online surveys were deployed annually to 2017, with another survey deployed in 2019.

### Participants

**Fig. 1:**
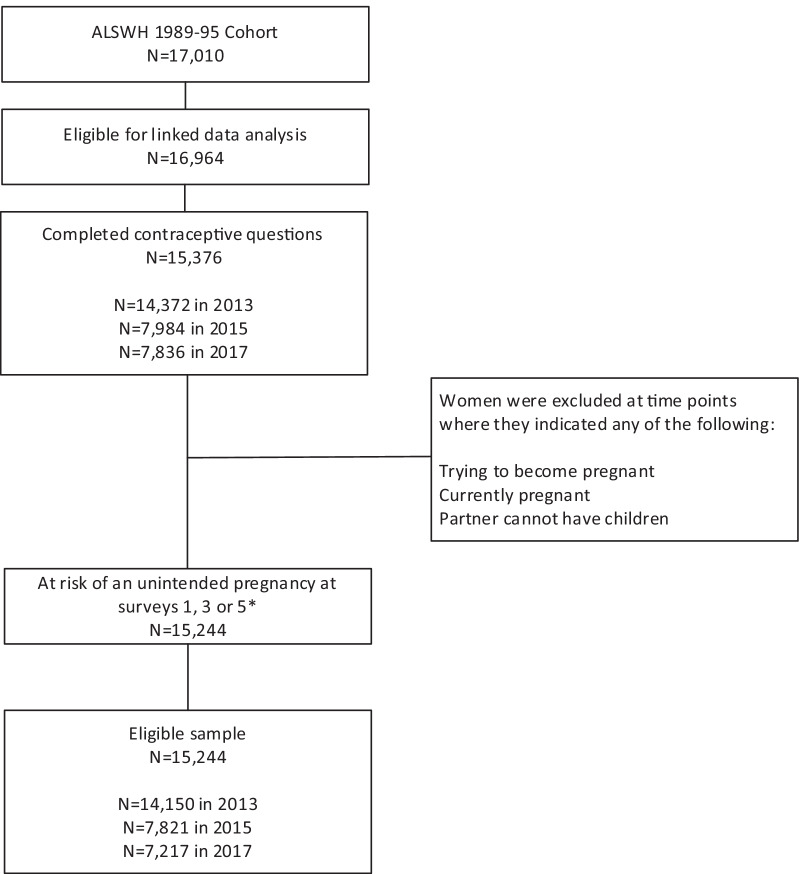
Determination of eligible sample. *Percentage of women at each survey who completed contraceptive questions but were excluded due to being not at risk of an unintended pregnancy: 2013 = 1.5%, 2015 = 2.0%, 2017 = 7.9%

This analysis focused on women from the 1989–1995 cohort who completed Surveys 1, 3 or 5 conducted in 2012–2013 (aged 18–24 years), 2015 (aged 20–26 years) and 2017 (aged 22–28 years). Of the 17,010 women who completed the baseline survey, 15,376 women were eligible for linked data analysis and completed the questions related to contraceptive use (Fig. 1). Women were considered not at risk of an unintended pregnancy at each time point if they reported being currently pregnant, trying to become pregnant, or if their partner could not have children. Based on these criteria, 132 women were not at risk of a future unintended pregnancy at all three time points, resulting in a final sample of 15,244 women (90% of the original cohort).

### Measures

#### Contraceptive use

Given the dynamic nature of contraceptive use, it was assessed at each survey. Participants were asked to report their contraceptive use the last time they had vaginal sex from a list of six options: “the pill”; “condoms”; “Implanon” (i.e., progestogen-only implant); “Mirena” (i.e., progestogen IUD); “other contraceptive”; and “none”. At Survey 1, women were also provided the opportunity to expand on their response to “other contraceptive” as part of a free-text option. For the purposes of analysis, the progestogen-only implant and progestogen IUD were combined into a single “hormonal LARC” item. Further, given the very low prevalence of the copper IUD in the free-text responses at Survey 1 (n = 24), this method was included as ‘other contraceptive’ at follow-up surveys.

#### Chronic disease

The presence or absence of seven physical chronic diseases that have been found to be relatively common among women of reproductive age and have been associated with poor maternal and perinatal outcomes were examined at each time point [4]. These included diabetes, cardiac disease (including hypertension), asthma, autoinflammatory arthropathies and connective tissue disease, IBD, multiple sclerosis, and thyroid disease. Disease ascertainment was assessed using multiple data sources and employing disease-specific algorithms (developed in concert with clinical experts) to increase chronic disease accuracy. Detailed information on the methods employed are described elsewhere [14]. Briefly, case ascertainment was achieved using survey and linked administrative health data, including individual state and territory-based Admitted Patient Data Collections (APDC) for hospital admissions; the Pharmaceutical Benefits Scheme (PBS) for prescribed medications, and the Medicare Benefits Schedule (MBS) for disease-specific medical claims. “Cases” were required to have either (a) one or more indication(s) in either the APDC or MBS; (b) indication in two or more ALSWH surveys; or (c) two or more disease-related prescriptions within a 12-month period, reported in two separate calendar years. Where a medication had multiple indications for treatment, medication ascertainment was used if there was an indication for that condition from another data source (e.g., was also captured through MBS or survey data).

#### Covariates

Time-varying covariates were assessed at each survey. Sociodemographic variables included age (years), highest educational qualification (year 12 or below; certificate/diploma; university), area of residence (categorised according to the Accessibility/Remoteness Index of Australia [ARIA+] classification system as major cities; inner regional; outer regional/remote/very remote), relationship status (partnered; unpartnered), employment status (full-time; part-time; not in paid work), country of birth (Australia; other English speaking; other), and ability to manage on available income (impossible/difficult always; difficult sometimes; not too bad/easy). Possession of a Health Care Card (a concession card for government-subsidised health care) was also included as a surrogate for socioeconomic status (yes; no).

Health-related factors included smoking status (current smoker; non-smoker), alcohol consumption (non-drinker; low risk drinker; infrequent drinker; risky/high risk drinker) [19] and body mass index (BMI: underweight [< 18.5 kg/m^2^]; healthy weight [≥ 18.5 and < 25 kg/m^2^]; overweight [≥ 25 and < 30 kg/m^2^]; obese [≥ 30 kg/m^2^]) [20]. Psychological distress was measured using the Kessler 10 (K10) scale with scores aggregated into categories (low [scores 10–15]; moderate [scores 16–21]; high [scores 22–29]; very high [scores 30–50]) [21].

Reproductive health factors included pregnancy history (yes; no), history of pregnancy termination (yes; no) and history of miscarriage (yes; no). Given contraceptive methods are often used for non-contraceptive reasons, we also adjusted for the presence or absence of self-reported gynaecological conditions such as polycystic ovarian syndrome (yes; no), endometriosis (yes; no), and the experience of menstrual symptoms such as irregular periods, heavy period, or severe period pain ‘often’ (yes; no).

### Statistical analysis

Latent transition analysis was used to group women into latent statuses over time based on reported contraceptive combinations [15]. The latent statuses relate to multiple types of contraceptives being used concurrently. For example, the latent status named ‘Pill and condom’ refers to women who used pills and condoms simultaneously. Women could transition between latent statuses over time, and the probabilities of these transitions are presented in Additional file 1: Table S3. Women appeared in the analysis for each survey they completed where they were at risk of an unintended pregnancy, up to a maximum of three times. They were assigned a latent status at each survey, which could be the same or vary over time.

Contraceptive data were entered into separate latent transition models with three to eight latent statuses each to determine the combinations of contraceptive use that best fit the data. A classify-analyse approach was used to assign each participant to a latent status at each time point, according to the latent status with the greatest posterior probability. Latent transition analysis was performed using PROC LTA procedure (The Methodology Centre, Penn State) in SAS 9.4 software. Multinomial mixed-effect models using generalised structural equation modelling was then developed in Stata 15.1, with the assigned latent status describing a particular contraceptive behaviour as the multinomial outcome, and time-varying predictors (including an indicator for chronic disease). Correlation between observations was accounted for by treating participant ID as a random intercept. Separate models were conducted for the presence of any chronic disease and each of the chronic diseases. We performed a complete case analysis with participants with missing data omitted from the analysis.

## Results

### Sample characteristics

At baseline in 2013, 18.9% of women reported having at least one of the physical chronic diseases of interest, and by 2017, this had increased to 22.6% (Table 1). The most prevalent chronic disease reported at baseline was asthma (14.3%), with the prevalence of diabetes, cardiac disease, autoinflammatory arthropathies and connective tissue disease, IBD, multiple sclerosis and thyroid disease all less than 5%. By 2017, the prevalence of asthma had increased to 16.1%, and slight increases were observed for all other conditions. Given the low frequencies of autoinflammatory conditions (i.e., autoinflammatory arthropathies and connective tissue disease, IBD, multiple sclerosis, and thyroid disease), these were combined into a single autoinflammatory disease category for subsequent modelling.

**Table 1:** Prevalence of chronic disease over time (2013–2017) among Australian women born 1989–1995

Chronic disease	Survey 1 (2013) Aged 18–24		Survey 3 (2015) Aged 20–26		Survey 5 (2017) Aged 22–28	
	n	%	n	%	n	%
Any chronic disease	2674	18.9	1707	21.8	1628	22.6
Diabetes	388	2.7	289	3.7	282	3.9
Cardiac disease	277	2.0	206	2.6	208	2.9
Asthma	2028	14.3	1238	15.8	1160	16.1
Autoinflammatory arthropathies	179	1.3	122	1.6	116	1.6
Inflammatory bowel disease	56	0.4	48	0.6	52	0.7
Thyroid disease	145	1.0	112	1.4	122	1.7
Multiple sclerosis	13	0.1	18	0.2	22	0.3
Autoinflammatory disease^a^	405	2.9	302	3.9	297	4.1

Number of women in 2013 (N = 14,150); 2015 (N = 7821); 2017 (N = 7217)

^a^Includes autoinflammatory arthropathies, inflammatory bowel disease, thyroid disease and multiple sclerosis

At baseline in 2013 (aged 18–24 years), there were few differences in characteristics between women with and without physical chronic disease, except for income management, BMI, and the experience of menstrual symptoms (Table 2). Women with chronic disease were more likely to report their ability to manage on available income as impossible or difficult always (30.7% vs. 25.4%). Women with chronic disease were also less likely to report being in the healthy BMI category than women without chronic disease (52.5% vs 60.0%) and were more likely to report menstrual symptoms often (42.6% vs 35.9%).

**Table 2:** Baseline characteristics of Australian women born 1989–1995 (when aged 18–24 in 2013), according to chronic disease status (n = 14,150)

Characteristic	Category	Presence of chronic disease	
		No n = 11,476 n (%)	Yes n = 2674 n (%)
Sociodemographics			
Country of birth	Australia	10,472 (91.3)	2512 (93.9)
	Other English-speaking background	414 (3.6)	71 (2.7)
	Non-English-speaking background	434 (3.8)	65 (2.4)
	*Missing*	*156 (1.4)*	*26 (1.0)*
Area of residence	Major cities	8616 (75.1)	1960 (73.3)
	Inner regional	1935 (16.9)	514 (19.2)
	Outer regional/remote/very remote	921 (8.0)	199 (7.4)
	*Missing*	*4 (0.0)*	*1 (0.0)*
Education	Year 12 or below	5615 (48.9)	1286 (48.1)
	Certificate/diploma	3238 (28.2)	817 (30.6)
	University	2616 (22.8)	571 (21.4)
	*Missing*	*7 (0.1)*	*0 (0.0)*
Relationship status	Partnered	3572 (31.1)	914 (34.2)
	Non-partnered	7898 (68.8)	1759 (65.8)
	*Missing*	*6 (0.1)*	*1 (0.0)*
Work status	Not in paid employment	2291 (20.0)	642 (24.0)
	Part-time	6233 (54.3)	1401 (52.4)
	Full-time	2939 (25.6)	630 (23.6)
	*Missing*	*13 (0.1)*	*1 (0.0)*
Income management	Impossible/difficult always	2912 (25.4)	821 (30.7)
	Difficult sometimes	4162 (36.3)	1000 (37.4)
	Not too bad/easy	4393 (38.3)	851 (31.8)
	*Missing*	*9 (0.1)*	*2 (0.1)*
Health care card status	No	*7838 (68.3)*	*1638 (61.3)*
	Yes	3631 (31.6)	1035 (38.7)
	*Missing*	*7 (0.1)*	*1 (0.0)*
Health factors			
Smoking status	Non-smoker	9063 (79.0)	2084 (77.9)
	Current smoker	2413 (21.0)	590 (22.1)
	*Missing*	*0 (0)*	*0 (0)*
Alcohol consumption	Non-drinker	563 (4.9)	175 (6.5)
	Low risk drinker	6813 (59.4)	1427 (53.4)
	Infrequent drinker	3621 (31.6)	964 (36.1)
	Risky/high risk drinker	479 (4.2)	108 (4.0)
	*Missing*	*0 (0)*	*0 (0)*
Body mass index	Underweight	904 (7.9)	152 (5.7)
	Healthy weight	6882 (60.0)	1403 (52.5)
	Overweight	2080 (18.1)	570 (21.3)
	Obese	1307 (11.4)	486 (18.2)
	*Missing*	*303 (2.6)*	*63 (2.4)*
Psychological distress (K10)	Low	2474 (21.6)	445 (16.6)
	Moderate	3380 (29.5)	716 (26.8)
	High	3152 (27.5)	787 (29.4)
	Very high	2469 (21.5)	726 (27.2)
	*Missing*	*1 (0.0)*	*0 (0.0)*
Reproductive health			
History of pregnancy	No	9883 (86.1)	2211 (82.7)
	Yes	1589 (13.8)	463 (17.3)
	*Missing*	*4 (0.1)*	*0 (0)*
History of termination	No	10,696 (93.2)	2481 (92.8)
	Yes	765 (6.7)	191 (7.1)
	*Missing*	*15 (0.1)*	*2 (0.1)*
History of miscarriage	No	10,938 (95.3)	2494 (93.3)
	Yes	521 (4.5)	177 (6.6)
	*Missing*	*17 (0.1)*	*3 (0.1)*
Parity	Zero	11,072 (96.5)	2517 (94.1)
	One	312 (2.7)	115 (4.3)
	Two	76 (0.7)	37 (1.4)
	Three or more	16 (0.1)	5 (0.2)
	*Missing*	*0 (0.0)*	*0 (0.0)*
Menstrual symptoms	No	7353 (64.1)	1536 (57.4)
	Yes	4123 (35.9)	1138 (42.6)
	*Missing*	0 (0)	*0 (0)*
History of PCOS	No	11,116 (96.9)	2489 (93.1)
	Yes	360 (3.1)	185 (6.9)
	*Missing*	*0 (0)*	*0 (0)*
History of endometriosis	No	11,224 (97.8)	2573 (96.2)
	Yes	252 (2.2)	101 (3.8)
	*Missing*	*0 (0)*	*0 (0)*

*PCOS* Polycystic ovary syndrome

### Trends in contraceptive use

In 2013, the proportion of women using some form of contraception at the time of their last vaginal sex was similar for women with (85.5%) and without chronic disease (86.7%) (Table 3), with similar proportions observed in 2017. By 2017, there was lower use of the oral contraceptive pill and condoms, although use of hormonal LARC had increased, with a noticeable increase in the use of the progestogen IUD. In 2013, use of the progestogen IUD was relatively low for women with and without chronic disease (3.0% vs. 1.8% respectively) but had a similar rise in both groups of women by 2017 (10.8% vs. 8.8% respectively).

**Table 3:** Observed contraceptive use over time (2013–2017) among Australian women born 1989–1995, according to chronic disease status

Contraceptive	Chronic disease status 2013 (Survey 1, aged 18–24)		Chronic disease status 2015 (Survey 3, aged 20–26)		Chronic disease status 2017 (Survey 5, aged 22–28)	
	No n = 11,476 n (%)	Yes n = 2674 n (%)	No n = 6114 n (%)	Yes n = 1707 n (%)	No n = 5589 n (%)	Yes n = 1628 n (%)
Any contraception	10,108 (88.1)	2339 (87.5)	5450 (89.1%)	1536 (90.0%)	4953 (88.6)	1430 (87.8)
Pill	6343 (55.3)	1465 (54.8)	3292 (53.8%)	902 (52.8%)	2522 (45.1)	693 (42.6)
Condom	4950 (43.1)	1135 (42.4)	2433 (39.8%)	659 (38.6%)	2090 (37.4)	592 (36.4)
Progestogen-only implant	1188 (10.4)	283 (10.6)	712 (11.6%)	233 (13.6%)	671 (12.0)	202 (12.4)
Progestogen IUD	212 (1.8)	81 (3.0)	276 (4.5%)	83 (4.9%)	493 (8.8)	176 (10.8)
Other methods	68 (0.6)	10 (0.4)	217 (3.5%)	75 (4.4%)	247 (4.4)	106 (6.5)
No contraception	1368 (11.9)	335 (12.5)	664 (10.9%)	171 (10.0%)	636 (11.4)	198 (12.2)
Number of contraceptives						
0	1368 (11.9)	335 (12.5)	664 (10.9%)	171 (10.0%)	636 (11.4)	198 (12.2)
1	7244 (63.1)	1632 (61.0)	3990 (65.3%)	1126 (66.0%)	3897 (69.7)	1093 (67.1)
2	2832 (24.7)	701 (26.2)	1444 (23.6%)	405 (23.7%)	1045 (18.7)	336 (20.6)
3+	32 (0.3)	6 (0.2)	16 (0.3%)	5 (0.3%)	11 (0.2)	1 (0.1)

Types of contraception do not add to 100% due to being able to choose multiple contraceptive methods

Among women with chronic disease, 61.0% and 67.1% reported using only one contraceptive method in 2013 and 2017 respectively. The proportion of women who reported two or more contraceptive methods declined from one-quarter in 2013 to one-fifth in 2017. Around 12% of women reported not using any contraception in both 2013 and 2017, irrespective of chronic disease status.

### Contraceptive combinations

The optimal LTA model was selected based on clinical interpretability, latent class separation and G^2^, AIC and BIC (Additional file 1: Table S1). We also sought to minimise the number of time points with very low membership probabilities (< 2%) as this would contribute to numerical estimation issues in subsequent regression models. A six-status model was determined to be the optimal model for categorising complex contraceptive use, based on goodness-of-fit statistics and clear clinical interpretability. The six-status model was preferred over the five-status model, which exhibited two very similar latent statuses that both featured condoms and the pill. The seven-status model was unviable due to low status membership probabilities.

As presented in Table 4, the first status (described as “Condom”) was characterised by high condom use (100% probability) but included some supplementation with the oral contraceptive pill (6% probability). The second status (described as “Pill and condom”) was dominated by both the pill (91% probability) and condom use (94% probability). The third status (described as “Pill”) was dominated by use of the oral contraceptive pill (100% probability) with a low probability of condom use (10% probability). The fourth status, termed “LARC and condom”, included women predicted to use a hormonal LARC (100% probability) but also some condom supplementation (19% probability). The fifth status was termed “Other and condom”, which included women predicted to use other contraceptive methods (100% probability) and/or condoms (18% probability), while the sixth status (described as “None”) captured the absence of any active contraceptive methods. Status 4 (‘Pill’) was selected as the reference status as it has traditionally been the most popular contraceptive choice for young women. This was also reflected in the data, with Status 4 the most common latent status across all three time points. Women had approximately a one in three probability of being in the ‘Pill’ latent status (Additional file 1: Table S2). For this reason, the pill made sense as a baseline contraceptive against which to make comparisons.

**Table 4:** Probability of individual contraception contributing to contraceptive patterns over time for Australian women born 1989–1995, using a six-status LTA model

Latent status	Latent status description (contraceptive pattern)	Item-response probabilities for each status				
		Condom	Pill	LARC^a^	Other	None
Status 1	Condom	1.00	0.06	–	–	–
Status 2	Pill and condom	0.91	0.94	–	–	
Status 3	None	–	–	–	–	0.95
Status 4	Pill	0.10	1.00	–	–	–
Status 5	LARC^a^ and condom	0.19	0.03	1.00	–	–
Status 6	Other and condom	0.18	0.04	–	1.00	–

Dashed cells have probability < 0.01

^a^LARC refers to the use of hormonal long-acting reversible contraception (progestogen-only implant and the progestogen IUD)

### Contraceptive patterns over time

Women were most likely to remain in the same contraceptive latent status between 2013, 2015, and 2017 (Additional file 1: Table S3). However, there was also substantial movement between contraceptive latent statuses over time. For example, there was a 35% probability that women using other contraceptive and condoms in 2013 would be using no contraception in 2015. Women using no contraception in 2013 were equally likely to transition to LARC and condoms (P = 0.11), or other contraception and condoms (P = 0.10) when measured again in 2015.

### Contraceptive use by women with chronic disease

Following the adjustment for confounders, the presence of any chronic disease was associated with increased odds of using other contraception and condoms (OR = 1.29, 95% CI 1.07 to 1.57), compared to use of the pill alone (Table 5). When focused on the relationship between specific chronic diseases and contraceptive use over time, women with cardiac disease had increased odds of combined pill and condom use (OR = 1.39, 95% CI 1.03 to 1.89), as well as no contraception (OR = 1.54, 95% CI 1.10 to 2.16), compared to the use of the pill alone. Notably, there was more than a twofold increase in the odds of using other contraception and condoms observed for women with cardiac disease (OR = 2.20, 95% CI 1.34 to 3.59). Women with autoinflammatory disease had increased odds of LARC and condoms (OR = 1.58), increased odds of other contraception and condoms (OR = 1.69) and increased odds of combined pill and condom use (OR = 1.38) when compared to use of the pill alone. There was little evidence to suggest that contraceptive use was influenced by a diagnosis of diabetes or asthma.

**Table 5:** Multinomial mixed-effect models for the effect of chronic disease status on contraceptive use for Australian women, aged 18 to 28 across three time points (2013, 2015 and 2017)

Model	Chronic disease status	Condom OR (95% CI)	Pill and condom OR (95% CI)	^a^LARC and condom OR (95% CI)	Other and condom OR (95% CI)	None OR (95% CI)
1	Any physical chronic disease	0.90 (0.80, 1.02)	1.23 (1.02, 1.48)	1.14 (1.00, 1.29)	1.29 (1.07, 1.57)	0.92 (0.82, 1.02)
2	Cardiac disease	1.36 (0.97, 1.91)	1.39 (1.03, 1.89)	1.53 (0.92, 2.55)	2.20 (1.34, 3.59)	1.54 (1.10, 2.16)
3	Diabetes	0.86 (0.66, 1.12)	1.10 (0.73, 1.67)	1.06 (0.80, 1.41)	1.18 (0.78, 1.79)	0.95 (0.75, 1.21)
4	Asthma	0.89 (0.78, 1.02)	1.15 (0.93, 1.41)	1.04 (0.90, 1.20)	1.22 (0.98, 1.51)	0.88 (0.78, 1.00)
5	Autoinflammatory disease	0.90 (0.67, 1.19)	1.38 (1.09, 1.75)	1.58 (1.04, 2.41)	1.69 (1.11, 2.57)	1.03 (0.77, 1.38)

Reference latent status = Status 4 (“Pill”); reference level for chronic disease status = disease not present

Each model controlled for age, country of birth, area of residence, highest educational qualification, work status, managing on available income, smoking status, alcohol consumption, body mass index, psychological distress, history of pregnancy, history of termination, history of miscarriages, menstrual symptoms, history of polycystic ovary syndrome, history of endometriosis, and survey wave

^a^LARC refers to the use of hormonal long-acting reversible contraception (progestogen-only implant and the progestogen IUD). Copper IUD is included as part of “other” contraception

## Discussion

Young women with chronic disease were more likely to engage in the use of low efficacy contraception over the observation period compared to women in the general population. When specific chronic diseases were examined, use of low efficacy contraception was evident for women with cardiac and autoinflammatory conditions but not those with diabetes or asthma. This study provides insight into how young Australian women with chronic disease ‘actually’ use contraception at the population level. These findings have important implications for the delivery of contraceptive counselling and reproductive life planning for young women with chronic disease, especially those with cardiac and autoinflammatory diseases.

Overall contraceptive use among young women meeting our chronic disease definitions was found to be high across the 5-year observation period (above 85%). We found self-reported hormonal-based contraceptive use among women with chronic disease to be substantially higher than that reported by DeNoble et al. [12]. While our prevalence of contraceptive use among women with chronic disease was similar to that reported in a 2016 cross-sectional U.S. study, they found that the use of effective and highly effective contraception was lowest among women aged 18–24 years (< 45%) with highly effective contraceptive use driven largely by sterilisation (even for young women) [13]. In contrast, high efficacy contraceptive use in our study was attributed to the use of hormonal LARC. This finding is important given that LARC use has previously been reported as low among young women in Australia [10, 15, 22] although more recent evidence suggests that hormonal LARC use is much higher among young women [23]. Our finding may be reflective of increased awareness in Australia around the acceptability of LARC across the reproductive life course and in particular the suitability of LARC for young nulliparous women. Increased LARC use is now also recognised as a key indicator in meeting the priorities of the current Australian National Women’s Health Strategy 2020–2030 [24].

Although our findings demonstrate that LARC use is increasing among young women, when we examined patterns of contraceptive use, only women diagnosed with autoinflammatory disease were found to have 58% increased odds of hormonal LARC use compared to women without chronic disease using the pill. While this finding is promising given that LARC are recommended as first line options for women with autoinflammatory disease (including those on immunosuppressants) and provide the greatest protection against unintended pregnancy [9, 25], they contrast with a number of studies which have demonstrated low LARC uptake among this chronic disease population [26, 27]. Concerningly, while women with autoinflammatory conditions were more likely to use LARC than other women, they also had a 69% increase in odds of using low efficacy methods compared to women without chronic disease using the pill. Our finding is supported by research showing that women with SLE discontinue hormonal contraception (mainly the combined oral contraceptive pill) following diagnosis and take up lower efficacy methods despite being on potentially teratogenic medications (including methotrexate) [28]. Use of low efficacy methods with high typical use failure rates place these women at significant risk of unintended pregnancy. Withdrawal and condoms have been found to be the most prevalent forms of contraception used by women with SLE and RA including a substantial number with unintended pregnancy histories [29]. Most concerning, however, is that young women diagnosed with cardiac disease had 220% and 54% increased odds of using low efficacy methods and of being non-users of contraception, compared to women without chronic disease using the pill, respectively. This finding is supported by previous cross-sectional research [6, 13]. Our findings, coupled with the emerging body of literature around contraceptive use among cardiac and autoinflammatory disease point to an unmet need regarding evidence-based contraceptive advice and support, particularly from rheumatologists and cardiologists who are responsible for prescribing (potentially teratogenic) medication and monitoring disease activity.

In our study no discernible difference in the contraceptive patterns between women with diabetes compared to women without chronic disease using the pill were found. While the international evidence around this topic has been equivocal, Australian research has found that although women with diabetes are high users of contraception (mostly condoms and the oral contraceptive pill), contraception is not consistently used [30]. This is important given that the pill and condoms are the most prevalent forms of contraception used at the time of unintended pregnancy among young Australian women [31]. Therefore, although the combined oral contraceptive pill is not contraindicated for young women with uncomplicated diabetes, increasing the use of highly effective contraception among this population is still warranted given the need for engagement in preconception contraceptive care to prevent adverse maternal and perinatal consequences associated with unintended pregnancy [32]. International research has shown only 32% of teens and 18% of young adults with type 1 diabetes attain recommended glycaemic control, yet only one-quarter of adolescents are aware of the adverse impacts associated with poor glycaemic control in pregnancy [33].

While young women with chronic disease should be supported to choose and use a method of contraception that aligns with their reproductive and personal goals, our findings point to an underutilisation of highly effective LARC among most young women with chronic disease and suggest that gaps in the delivery of preconception contraceptive counselling may exist in Australia, particularly for those with cardiac and autoinflammatory conditions. Low rates of general and disease-specific contraceptive counselling among chronic disease populations have been demonstrated internationally, even in the presence of potentially teratogenic medication [34–36]. The reasons for low contraceptive counselling among women with chronic disease are not well understood. It has been postulated that lack of both contraceptive use and contraceptive counselling among women with chronic disease is attributed to misperceptions around fertility, knowledge regarding pregnancy risks [37, 38] and health system factors whereby the chronic condition takes up the health care providers’ time and focus during appointments [39]. As a result, women with chronic disease often receive minimal contraceptive counselling from either general practitioners (GPs) or specialists. It has also been argued that health care providers are uncomfortable with prescribing contraception to women they perceive to be at higher risk of adverse events due to lack of familiarity with the safety of different methods for women with different medical conditions [40].

The contraceptive needs of women with cardiac disease are especially challenging to navigate due to variability in potential risks associated with both contraception type and the nature and severity of the cardiac disease. In general, however, medical eligibility guidelines for the provision of contraception [25], indicate the risks associated with the use of estrogen-containing hormonal contraception (e.g., combined oral contraceptive pill) outweigh the benefits given the increased risks of arterial and venous thrombosis for a number of cardiac conditions, including severe or poorly controlled hypertension (although in practice, the combined oral contraceptive pill is generally only advised against when there is a history of unprovoked arterial/venous thrombosis or a known genetic defect). By contrast, progestogen-only methods are not associated with a risk of venous or arterial thromboembolic disease and are safer options for most women with cardiac disease (including those with congenital heart disease) [41, 42]. Of the progestogen-only methods available, the progestogen-only implant followed by levonorgestrel-IUDs have the highest efficacy against pregnancy. Levonorgestrel IUDs are a suitable choice for women with cardiac conditions (including those with congenital heart disease), and their effect on reducing menstrual bleeding can be beneficial, including for those on anticoagulant therapy. However, women with major cardiac disease may require cardiology input before insertion [25]. Similarly, in the presence of autoinflammatory conditions such as RA and IBD, there are concerns associated with estrogen-containing contraception in relation to disease exacerbation as well as thrombotic effects (particularly with women who have antiphospholipid syndrome and a history of IBD-related surgery or past biologic therapy use). Potential malabsorption issues for women with IBD will also limit the use of combined hormonal and progestogen-only oral methods.

Given the exposure to medications used in the treatment of autoinflammatory conditions, chronic hypertension and other cardiac diseases are associated with major congenital malformations [43–45], young women with chronic disease (particularly those with cardiac and autoinflammatory conditions) therefore require individualised contraceptive counselling and reproductive life planning based on their specific condition(s), with consideration around disease severity and medication use [25]. As these women are already engaged within the healthcare system this provides an ideal opportunity to provide such care as part of a well-coordinated structured approach to chronic disease management involving GPs and specialists where contraceptive conversations are routine. While some key bodies such as the Australian Rheumatology Association provide guidance on prescribing medications during pregnancy and recommend that women of child-bearing age receive preconception counselling and discussions around contraception, Australia currently lacks formal guidelines for autoinflammatory diseases as well as other chronic conditions. Increased access to, and awareness of current therapeutic guidelines by peak medical associations and key bodies (e.g., Therapeutic Guidelines) as well as development of referral pathways are required alongside increasing medical education given there is a demonstrated lack of expertise and confidence regarding the provision of family planning among GPs and specialists in Australia and internationally [46–48]. This will ensure that young women with chronic disease receive access to clear and accurate information regarding their contraceptive options. Increased education for young women with chronic disease about the risks of unintended pregnancy and the benefits of appropriate highly effective contraception for their specific condition, as well as evidence-based information to dispels myths around LARC including its impact on future fertility are also required [49]. Importantly for young women who develop chronic disease early in life, greater acknowledgement, information provision and screening of paediatric populations in relation to sexual activity and contraceptive needs is needed [50]. Such practices also need to be maintained as young women transition from paediatric to adult services to ensure these women do not fall through the gap.

A key strength of this study was the ability to examine contraceptive patterns for women diagnosed with seven key chronic diseases during early adulthood using longitudinal data. In addition, we were able to apply statistical techniques to accurately identify contraceptive combinations. This improves on previous research which has examined contraception within the context of chronic disease as either users or non-users or has used hierarchical approaches [12, 13]. Our analysis shows that for young women contraceptive use is complex and requires examination of all contraceptive combinations. A further strength of the study is the methods used to ascertain chronic disease cases with the inclusion of both survey and administrative data providing the ability to capture all forms of chronic disease [14]. No studies have previously utilised these approaches for examining chronic disease among women of reproductive age. Given that contraceptive use and risk of an unintended pregnancy is dynamic across the reproductive life course [16], we excluded women not at risk of a future unintended pregnancy at each of the time points. Few studies have accounted for this in longitudinal research [51].

While we were able to examine contraceptive use at yearly intervals, the questions regarding contraceptive use were not substantive after survey 1. Unfortunately, we were not able to accurately ascertain the use of methods such as the depot injection or copper IUD. Due to minimal self-reports of their use, these items were included as ‘other’ contraception. As such, we have potentially over-estimated the use of low efficacy methods. Also, there may have been some bias in the sample due to differential loss to follow-up. It is unclear whether women with chronic disease would be more, or less, likely to complete surveys. Additionally, while we examined contraception over time using latent transition analysis, to examine time-varying covariates (including chronic disease status) we employed a classify-analyse approach. We acknowledge that this approach may induce some measurement error due to the uncertainty in latent status classification [52].

## Conclusion

This study demonstrated that young Australian women with chronic disease take up contraception at similar rates to their same aged peers in the community, although they are more likely to use less effective contraception, particularly women with cardiac and autoinflammatory diseases. Our findings highlight the need for all young women with chronic disease to have the opportunity for comprehensive contraceptive counselling as part of their routine chronic disease management, at diagnosis and at regular ongoing appointments, to ensure they are aware of the risks of unintended pregnancy and are provided with the highest efficacy contraceptive options that are most appropriate to their specific circumstances. To achieve this, increasing contraceptive knowledge and awareness of the need for contraceptive counselling among specialists and improvement in communication between GPs and specialists as part of a well-coordinated teams-based approach to chronic disease management is required. This will not only increase women’s agency in contraceptive knowledge and choices but also reduce high-risk unintended pregnancies in this vulnerable population.

## Supplementary Information

**Additional file 1: Table S1.** Summary of selected LTA model diagnostics. **Table S2.** Predicted prevalence of each latent status (delta estimates) over time, for a six-status LTA model. **Table S3.** Latent status transition probabilities (tau estimates) from Time 1 (2013) to Time 2 (2015), and from Time 2 (2015) to Time 3 (2017). **Table S4.** Full multinomial mixed-effect models for factors associated with contraceptive use by chronic disease status (any chronic disease) for the ALSWH 1989–1995 cohort. **Table S5.** Full multinomial mixed-effect model for factors associated with contraceptive use by cardiac disease status for the ALSWH 1989–1995 cohort. **Table S6.** Full multinomial mixed-effect model for factors associated with contraceptive use by diabetes status for the ALSWH 1989–1995 cohort. **Table S7.** Full multinomial mixed-effect model for factors associated with contraceptive use by asthma status for the ALSWH 1989–1995 cohort. **Table S8.** Full multinomial mixed-effect model for factors associated with contraceptive use by autoinflammatory disease status for the ALSWH 1989–1995 cohort.

## Data Availability

The data used as part of this analysis are bound by ethical restrictions due to containing person level data. These restrictions have been imposed by the ALSWH data access committee. A core survey dataset is available through the Australian Data Archive (please visit https://dataverse.ada.edu.au/dataverse/alswh_core_release for further information about obtaining these data). For further information regarding full access to Australian Longitudinal Study on Women’s Health data, including linked datasets, requests are to be sent to info@alswh.org.au.

## Abbreviations

RA: Rheumatoid arthritis
IBD: Inflammatory bowel disease
SLE: Systemic lupus erythematosus
ALSWH: Australian Longitudinal Study on Women’s Health
APDC: Admitted Patient Data Collection
MBS: Medical Benefit Schedule
PBS: Pharmaceutical Benefits Scheme
BMI: Body Mass Index
LARC: Long-acting reversible contraception
GP: General practitioner
IUD: Intrauterine device
