# Supplementary material for: Patterns of contraceptive use among young Australian women with chronic disease: findings from a prospective cohort study
Source: Reprod Health. 2022 May 7;19:111. doi: 10.1186/s12978-022-01413-x (PMC9078003; doi:10.1186/s12978-022-01413-x)
Supplement: Supplementary file 1 — Additional file 1: Table S1. Summary of selected LTA model diagnostics. Table S2. Predicted prevalence of each latent status (delta estimates) over time, for a six-status LTA model. Table S3. Latent status transition probabilities (tau estimates) from Time 1 (2013) to Time 2 (2015), and from Time 2 (2015) to Time 3 (2017). Table S4. Full multinomial mixed-effect models for factors associated with contraceptive use by chronic disease status (any chronic disease) for the ALSWH 1989–1995 cohort. Table S5. Full multinomial mixed-effect model for factors associated with contraceptive use by cardiac disease status for the ALSWH 1989–1995 cohort. Table S6. Full multinomial mixed-effect model for factors associated with contraceptive use by diabetes status for the ALSWH 1989–1995 cohort. Table S7. Full multinomial mixed-effect model for factors associated with contraceptive use by asthma status for the ALSWH 1989–1995 cohort. Table S8. Full multinomial mixed-effect model for factors associated with contraceptive use by autoinflammatory disease status for the ALSWH 1989–1995 cohort. [file 12978_2022_1413_MOESM1_ESM.docx]

**Additional file**

**Table S1. Summary of selected LTA model diagnostics**

| **Number of latent statuses** | **Number of iterations** | **G^2^** | **AIC** | **BIC** |
| --- | --- | --- | --- | --- |
| 3 | 144 | 14183.03 | 14241.03 | 14462.36 |
| 4 | 146 | 12321.86 | 12415.86 | 12774.56 |
| 5 | 504 | 3091.52 | 3229.52 | 3756.12 |
| 6 | 1652 | 1635.63 | 1825.63 | 2550.66 |
| 7 | 18609 | 1578.66 | 1828.66 | 2782.65 |
| 8 | 7560 | 1194.27 | 1512.27 | 2725.75 |

**Table S2. Predicted prevalence of each latent status (delta estimates) over time, for a six-status LTA model.**

| **Latent Status** | **Latent status description** | **Time 1**  **(2013)**  **(%)** | **Time 2**  **(2015)**  **(%)** | **Time 3**  **(2017)**  **(%)** |
| --- | --- | --- | --- | --- |
| Status 1 | Condom | 19 | 17 | 20 |
| Status 2 | Pill and condom | 21 | 16 | 10 |
| Status 3 | None | 13 | 12 | 13 |
| Status 4 | Pill | 34 | 35 | 31 |
| Status 5 | ^a^LARC and condom | 12 | 16 | 21 |
| Status 6 | Other and condom | 1 | 4 | 5 |

^a^ LARC refers to the use of hormonal long-acting reversible contraception (progestogen-only implant and the progestogen IUD).

**Table S3. Latent status transition probabilities (tau estimates) from Time 1 (2013) to Time 2 (2015), and from Time 2 (2015) to Time 3 (2017).**

| **Time 1 (2013) to Time 2 (2015)** | | | | | | | | |
| --- | --- | --- | --- | --- | --- | --- | --- | --- |
|  |  | Time 2 (2015, aged 20-26) | | | | | | |
|  |  | Condom | Pill & condom | None | Pill | LARC & condom | Other & condom |  |
| Time 1 (2013, aged 18-24) | Condom | 0.51† | 0.05 | 0.12 | 0.18 | 0.09 | 0.05 |  |
|  | Pill & condom | 0.08 | 0.61† | 0.03 | 0.17 | 0.09 | 0.02 |  |
|  | None | 0.17 | 0.02 | 0.41† | 0.19 | 0.11 | 0.10 |  |
|  | Pill | 0.08 | 0.05 | 0.07 | 0.70† | 0.08 | 0.02 |  |
|  | LARC & condom | 0.07 | 0.02 | 0.06 | 0.11 | 0.70† | 0.04 |  |
|  | Other & condom | 0.12 | 0.09 | 0.35† | 0.17 | 0.04 | 0.22 |  |
| **Time 2 (2015) to Time 3 (2017)** | | | | | | | | |
|  |  | Time 3 (2017, aged 22-28) | | | | | | |
|  |  | Condom | Pill & condom | None | Pill | LARC & condom | Other & condom |  |
| Time 2 (2015, aged 20-26) | Condom | 0.55† | 0.03 | 0.15 | 0.13 | 0.11 | 0.04 |  |
|  | Pill & condom | 0.11 | 0.59† | 0.01 | 0.16 | 0.11 | 0.02 |  |
|  | None | 0.21 | 0.01 | 0.48† | 0.12 | 0.12 | 0.06 |  |
|  | Pill | 0.12 | 0.01 | 0.08 | 0.65† | 0.11 | 0.03 |  |
|  | LARC & condom | 0.08 | 0.00 | 0.06 | 0.10 | 0.72† | 0.04 |  |
|  | Other & condom | 0.13 | 0.03 | 0.12 | 0.18 | 0.17 | 0.39† |  |
| † transition probability ≥ 0.30 | | | | | | | | |

**Table S4.** **Full multinomial mixed-effect models for factors associated with contraceptive use by chronic disease status (any chronic disease) for the ALSWH 1989-95 cohort.**

|  | **Condom** | | | **Pill & condom** | | | **LARC & condom** | | | **Other & condom** | | | **None** | | |
| --- | --- | --- | --- | --- | --- | --- | --- | --- | --- | --- | --- | --- | --- | --- | --- |
| **Factor** | **OR** | **95% CI** | ***P*** | **OR** | **95% CI** | ***P*** | **OR** | **95% CI** | ***P*** | **OR** | **95% CI** | ***P*** | **OR** | **95% CI** | ***P*** |
| Any physical chronic disease |  |  |  |  |  |  |  |  |  |  |  |  |  |  |  |
| No | Ref. | - | - | Ref. | - | - | Ref. | - | - | Ref. | - | - | Ref. | - | - |
| Yes | 0.90 | 0.80 - 1.02 | 0.09 | 1.23 | 1.02 - 1.48 | 0.03 | 1.14 | 1.00 - 1.29 | 0.05 | 1.29 | 1.07 - 1.57 | 0.01 | 0.92 | 0.82 - 1.02 | 0.12 |
| Age (years) | 0.99 | 0.96 - 1.02 | 0.44 | 0.85 | 0.81 - 0.90 | <0.001 | 0.94 | 0.91 - 0.97 | <0.001 | 1.07 | 1.01 - 1.13 | 0.02 | 1.05 | 1.03 - 1.09 | <0.001 |
| Country of birth |  |  |  |  |  |  |  |  |  |  |  |  |  |  |  |
| Australia | Ref. | - | - | Ref. | - | - | Ref. | - | - | Ref. | - | - | Ref. | - | - |
| Other ESB | 0.99 | 0.78 - 1.26 | 0.94 | 0.86 | 0.58 - 1.29 | 0.47 | 1.25 | 0.96 - 1.63 | 0.10 | 1.26 | 0.85 - 1.86 | 0.25 | 0.86 | 0.68 - 1.09 | 0.21 |
| Non-ESB | 1.65 | 1.32 - 2.08 | <0.001 | 0.63 | 0.42 - 0.94 | 0.02 | 0.94 | 0.70 - 1.25 | 0.65 | 1.49 | 0.99 - 2.24 | 0.06 | 1.55 | 1.25 - 1.94 | <0.001 |
| Area of residence |  |  |  |  |  |  |  |  |  |  |  |  |  |  |  |
| Major cities | Ref. | - | - | Ref. | - | - | Ref. | - | - | Ref. | - | - | Ref. | - | - |
| Inner regional | 0.94 | 0.83 - 1.06 | 0.33 | 1.16 | 0.97 - 1.40 | 0.10 | 1.10 | 0.96 - 1.26 | 0.15 | 0.86 | 0.68 - 1.10 | 0.23 | 0.94 | 0.84 - 1.06 | 0.30 |
| Outer regional/remote/very remote | 1.04 | 0.88 - 1.23 | 0.65 | 1.26 | 0.98 - 1.61 | 0.07 | 1.44 | 1.21 - 1.72 | <0.001 | 1.39 | 1.04 - 1.84 | 0.02 | 1.06 | 0.90 - 1.25 | 0.46 |
| Education |  |  |  |  |  |  |  |  |  |  |  |  |  |  |  |
| Year 12 or below | Ref. | - | - | Ref. | - | - | Ref. | - | - | Ref. | - | - | Ref. | - | - |
| Certificate/diploma | 0.95 | 0.85 - 1.07 | 0.41 | 0.91 | 0.77 - 1.08 | 0.29 | 1.11 | 0.98 - 1.26 | 0.09 | 0.97 | 0.78 - 1.21 | 0.78 | 1.07 | 0.96 - 1.19 | 0.24 |
| University | 0.94 | 0.84 - 1.06 | 0.31 | 1.16 | 0.98 - 1.37 | 0.09 | 1.09 | 0.96 - 1.24 | 0.18 | 0.72 | 0.57 - 0.90 | <0.001 | 0.68 | 0.60 - 0.77 | <0.001 |
| Relationship status |  |  |  |  |  |  |  |  |  |  |  |  |  |  |  |
| Partnered | Ref. | - | - | Ref. | - | - | Ref. | - | - | Ref. | - | - | Ref. | - | - |
| Unpartnered | 1.63 | 1.48 - 1.79 | <0.001 | 2.95 | 2.55 - 3.41 | <0.001 | 1.28 | 1.16 - 1.42 | <0.001 | 1.33 | 1.11 - 1.58 | <0.001 | 0.95 | 0.87 - 1.05 | 0.32 |
| Work status |  |  |  |  |  |  |  |  |  |  |  |  |  |  |  |
| Full-time | Ref. | - | - | Ref. | - | - | Ref. | - | - | Ref. | - | - | Ref. | - | - |
| None | 1.73 | 1.49 - 2.00 | <0.001 | 1.43 | 1.16 - 1.77 | <0.001 | 1.23 | 1.05 - 1.44 | 0.01 | 1.19 | 0.89 - 1.58 | 0.23 | 1.27 | 1.10 - 1.47 | <0.001 |
| Part-time | 1.32 | 1.19 - 1.46 | <0.001 | 1.44 | 1.24 - 1.66 | <0.001 | 1.07 | 0.96 - 1.20 | 0.21 | 0.91 | 0.75 - 1.11 | 0.36 | 0.99 | 0.89 - 1.10 | 0.80 |
| Income management |  |  |  |  |  |  |  |  |  |  |  |  |  |  |  |
| Not too bad/easy | Ref. | - | - | Ref. | - | - | Ref. | - | - | Ref. | - | - | Ref. | - | - |
| Difficult sometimes | 0.85 | 0.77 - 0.94 | <0.001 | 0.78 | 0.68 - 0.90 | <0.001 | 0.91 | 0.82 - 1.01 | 0.08 | 1.14 | 0.94 - 1.39 | 0.17 | 0.96 | 0.87 - 1.06 | 0.43 |
| Impossible/difficult always | 0.8 | 0.70 - 0.90 | <0.001 | 0.78 | 0.65 - 0.93 | 0.01 | 0.94 | 0.82 - 1.07 | 0.33 | 1.01 | 0.78 - 1.30 | 0.96 | 0.98 | 0.86 - 1.11 | 0.72 |
| Health care card status |  |  |  |  |  |  |  |  |  |  |  |  |  |  |  |
| No | Ref. | - | - | Ref. | - | - | Ref. | - | - | Ref. | - | - | Ref. | - | - |
| Yes | 0.95 | 0.86 - 1.06 | 0.36 | 0.95 | 0.82 - 1.10 | 0.52 | 1.18 | 1.06 - 1.33 | <0.001 | 1.02 | 0.81 - 1.27 | 0.88 | 1.01 | 0.91 - 1.13 | 0.79 |
| Smoking status |  |  |  |  |  |  |  |  |  |  |  |  |  |  |  |
| Non-smoker | Ref. | - | - | Ref. | - | - | Ref. | - | - | Ref. | - | - | Ref. | - | - |
| Current smoker | 1.06 | 0.94 - 1.19 | 0.35 | 0.45 | 0.37 - 0.55 | <0.001 | 1.09 | 0.96 - 1.25 | 0.17 | 1.09 | 0.88 - 1.36 | 0.43 | 1.68 | 1.51 - 1.87 | <0.001 |
| Alcohol consumption |  |  |  |  |  |  |  |  |  |  |  |  |  |  |  |
| Non-drinker | Ref. | - | - | Ref. | - | - | Ref. | - | - | Ref. | - | - | Ref. | - | - |
| Low risk drinker | 0.50 | 0.42 - 0.61 | <0.001 | 0.62 | 0.47 - 0.84 | <0.001 | 0.79 | 0.64 - 0.99 | 0.04 | 0.68 | 0.48 - 0.96 | 0.03 | 0.48 | 0.40 - 0.57 | <0.001 |
| Infrequent drinker | 0.60 | 0.49 - 0.73 | <0.001 | 0.69 | 0.51 - 0.92 | 0.01 | 0.80 | 0.64 - 1.00 | 0.05 | 0.67 | 0.47 - 0.96 | 0.03 | 0.66 | 0.55 - 0.80 | <0.001 |
| Risky/high risk drinker | 0.58 | 0.44 - 0.78 | <0.001 | 0.45 | 0.28 - 0.70 | <0.001 | 0.71 | 0.51 - 0.99 | 0.04 | 0.86 | 0.50 - 1.47 | 0.57 | 0.58 | 0.43 - 0.76 | <0.001 |
| Body Mass Index |  |  |  |  |  |  |  |  |  |  |  |  |  |  |  |
| Healthy weight | Ref. | - | - | Ref. | - | - | Ref. | - | - | Ref. | - | - | Ref. | - | - |
| Underweight | 1.03 | 0.86 - 1.22 | 0.77 | 0.81 | 0.62 - 1.06 | 0.12 | 0.78 | 0.63 - 0.97 | 0.02 | 0.72 | 0.46 - 1.12 | 0.14 | 1.13 | 0.95 - 1.35 | 0.17 |
| Overweight | 1.16 | 1.04 - 1.30 | 0.01 | 1.45 | 1.24 - 1.71 | <0.001 | 1.33 | 1.18 - 1.49 | <0.001 | 1.36 | 1.11 - 1.66 | <0.001 | 1.20 | 1.08 - 1.35 | <0.001 |
| Obese | 1.54 | 1.34 - 1.76 | <0.001 | 1.50 | 1.21 - 1.85 | <0.001 | 1.78 | 1.54 - 2.07 | <0.001 | 1.47 | 1.16 - 1.87 | <0.001 | 1.45 | 1.28 - 1.65 | <0.001 |
| Psychological distress (K10) |  |  |  |  |  |  |  |  |  |  |  |  |  |  |  |
| Low | Ref. | - | - | Ref. | - | - | Ref. | - | - | Ref. | - | - | Ref. | - | - |
| Moderate | 1.05 | 0.93 - 1.17 | 0.42 | 1.11 | 0.94 - 1.30 | 0.22 | 1.02 | 0.90 - 1.15 | 0.78 | 1.07 | 0.86 - 1.34 | 0.53 | 1.01 | 0.90 - 1.15 | 0.81 |
| High | 0.98 | 0.87 - 1.11 | 0.77 | 0.91 | 0.76 - 1.08 | 0.29 | 0.93 | 0.82 - 1.06 | 0.29 | 0.94 | 0.74 - 1.19 | 0.60 | 1.06 | 0.93 - 1.20 | 0.39 |
| Very high | 1.06 | 0.92 - 1.23 | 0.39 | 0.94 | 0.77 - 1.16 | 0.58 | 1.05 | 0.90 - 1.23 | 0.53 | 0.96 | 0.72 - 1.27 | 0.76 | 1.24 | 1.07 - 1.42 | <0.001 |
| Pregnancy history |  |  |  |  |  |  |  |  |  |  |  |  |  |  |  |
| N | Ref. | - | - | Ref. | - | - | Ref. | - | - | Ref. | - | - | Ref. | - | - |
| Yes | 2.70 | 2.17 - 3.37 | <0.001 | 0.67 | 0.45 - 1.00 | 0.05 | 2.77 | 2.19 - 3.51 | <0.001 | 2.80 | 1.99 - 3.95 | <0.001 | 4.09 | 3.39 - 4.93 | <0.001 |
| History of terminations |  |  |  |  |  |  |  |  |  |  |  |  |  |  |  |
| No | Ref. | - | - | Ref. | - | - | Ref. | - | - | Ref. | - | - | Ref. | - | - |
| Yes | 0.51 | 0.40 - 0.66 | <0.001 | 0.73 | 0.47 - 1.14 | 0.16 | 0.82 | 0.63 - 1.06 | 0.13 | 0.84 | 0.58 - 1.21 | 0.35 | 0.53 | 0.43 - 0.65 | <0.001 |
| History of miscarriage |  |  |  |  |  |  |  |  |  |  |  |  |  |  |  |
| No | Ref. | - | - | Ref. | - | - | Ref. | - | - | Ref. | - | - | Ref. | - | - |
| Yes | 0.65 | 0.50 - 0.84 | <0.001 | 1.16 | 0.74 - 1.84 | 0.52 | 0.59 | 0.45 - 0.79 | <0.001 | 1.01 | 0.69 - 1.48 | 0.94 | 0.97 | 0.79 - 1.20 | 0.79 |
| Menstrual symptoms |  |  |  |  |  |  |  |  |  |  |  |  |  |  |  |
| No | Ref. | - | - | Ref. | - | - | Ref. | - | - | Ref. | - | - | Ref. | - | - |
| Yes | 2.73 | 2.48 - 3.00 | <0.001 | 1.28 | 1.11 - 1.47 | <0.001 | 2.57 | 2.32 - 2.85 | <0.001 | 2.07 | 1.73 - 2.48 | <0.001 | 2.46 | 2.24 - 2.70 | <0.001 |
| History of PCOS |  |  |  |  |  |  |  |  |  |  |  |  |  |  |  |
| No | Ref. | - | - | Ref. | - | - | Ref. | - | - | Ref. | - | - | Ref. | - | - |
| Yes | 0.91 | 0.74 - 1.12 | 0.39 | 0.88 | 0.63 - 1.24 | 0.48 | 0.65 | 0.52 - 0.82 | <0.001 | 0.80 | 0.57 - 1.12 | 0.19 | 1.2 | 1.00 - 1.43 | 0.05 |
| History of endometriosis |  |  |  |  |  |  |  |  |  |  |  |  |  |  |  |
| No | Ref. | - | - | Ref. | - | - | Ref. | - | - | Ref. | - | - | Ref. | - | - |
| Yes | 0.73 | 0.55 - 0.97 | 0.03 | 1.50 | 0.98 - 2.29 | 0.06 | 1.89 | 1.43 - 2.48 | <0.001 | 1.20 | 0.80 - 1.80 | 0.37 | 0.80 | 0.63 - 1.03 | 0.08 |
| Time |  |  |  |  |  |  |  |  |  |  |  |  |  |  |  |
| Survey 1 | Ref. | - | - | Ref. | - | - | Ref. | - | - | Ref. | - | - | Ref. | - | - |
| Survey 3 | 0.89 | 0.80 - 0.99 | 0.03 | 0.82 | 0.71 - 0.95 | 0.01 | 1.33 | 1.18 - 1.49 | <0.001 | 5.69 | 4.31 - 7.51 | <0.001 | 0.70 | 0.63 - 0.79 | <0.001 |
| Survey 5 | 1.21 | 1.05 - 1.40 | 0.01 | 0.65 | 0.52 - 0.82 | <0.001 | 2.06 | 1.76 - 2.41 | <0.001 | 7.02 | 5.08 - 9.71 | <0.001 | 0.83 | 0.71 - 0.96 | 0.01 |

Notes: ESB = English-speaking background; Reference status = Status 4 (pill); A LARC here refers to the use of hormonal long-acting reversible contraception (progestogen-only implant and the progestogen

IUD). Parity was not included in the models due to overlap with pregnancy history.

**Table S5. Full multinomial mixed-effect model for factors associated with contraceptive use by cardiac disease status for the ALSWH 1989-95 cohort.**

|  | **Condom** | | | **Pill & condom** | | | **LARC & condom** | | | **Other & condom** | | | **None** | | |
| --- | --- | --- | --- | --- | --- | --- | --- | --- | --- | --- | --- | --- | --- | --- | --- |
| **Factor** | **OR** | **95% CI** | ***P*** | **OR** | **95% CI** | ***P*** | **OR** | **95% CI** | ***P*** | **OR** | **95% CI** | ***P*** | **OR** | **95% CI** | ***P*** |
| Cardiac disease |  |  |  |  |  |  |  |  |  |  |  |  |  |  |  |
| No | Ref. | - | - | Ref. | - | - | Ref. | - | - | Ref. | - | - | Ref. | - | - |
| Yes | 1.36 | 0.97 - 1.91 | 0.08 | 1.39 | 1.03 - 1.89 | 0.03 | 1.53 | 0.92 - 2.55 | 0.10 | 2.20 | 1.34 - 3.59 | <0.001 | 1.54 | 1.10 - 2.16 | 0.01 |
| Age (years) | 1.00 | 0.97 - 1.04 | 0.77 | 0.92 | 0.90 - 0.95 | <0.001 | 0.94 | 0.89 - 0.99 | 0.02 | 1.07 | 1.01 - 1.14 | 0.02 | 1.06 | 1.02 - 1.09 | <0.001 |
| Country of birth |  |  |  |  |  |  |  |  |  |  |  |  |  |  |  |
| Australia | Ref. | - | - | Ref. | - | - | Ref. | - | - | Ref. | - | - | Ref. | - | - |
| Other ESB | 1.04 | 0.80 - 1.36 | 0.76 | 0.96 | 0.76 - 1.21 | 0.72 | 1.41 | 0.92 - 2.14 | 0.11 | 1.32 | 0.86 - 2.03 | 0.21 | 0.91 | 0.69 - 1.21 | 0.52 |
| Non-ESB | 1.87 | 1.45 - 2.41 | <0.001 | 0.81 | 0.63 - 1.03 | 0.08 | 1.08 | 0.70 - 1.67 | 0.72 | 1.65 | 1.05 - 2.58 | 0.03 | 1.70 | 1.30 - 2.22 | <0.001 |
| Area of residence |  |  |  |  |  |  |  |  |  |  |  |  |  |  |  |
| Major cities | Ref. | - | - | Ref. | - | - | Ref. | - | - | Ref. | - | - | Ref. | - | - |
| Inner regional | 0.93 | 0.82 - 1.06 | 0.27 | 1.08 | 0.96 - 1.21 | 0.19 | 1.14 | 0.94 - 1.38 | 0.18 | 0.87 | 0.68 - 1.12 | 0.28 | 0.94 | 0.82 - 1.08 | 0.38 |
| Outer regional/remote/very remote | 1.05 | 0.88 - 1.26 | 0.59 | 1.23 | 1.05 - 1.44 | 0.01 | 1.55 | 1.20 - 2.00 | <0.001 | 1.43 | 1.06 - 1.94 | 0.02 | 1.10 | 0.91 - 1.33 | 0.31 |
| Education |  |  |  |  |  |  |  |  |  |  |  |  |  |  |  |
| Year 12 or below | Ref. | - | - | Ref. | - | - | Ref. | - | - | Ref. | - | - | Ref. | - | - |
| Certificate/diploma | 1.00 | 0.89 - 1.13 | 0.96 | 0.99 | 0.88 - 1.10 | 0.82 | 1.24 | 1.04 - 1.48 | 0.02 | 1.02 | 0.81 - 1.28 | 0.89 | 1.09 | 0.96 - 1.23 | 0.18 |
| University | 0.95 | 0.84 - 1.08 | 0.45 | 1.14 | 1.02 - 1.27 | 0.02 | 1.2 | 1.00 - 1.44 | 0.05 | 0.73 | 0.57 - 0.93 | 0.01 | 0.68 | 0.59 - 0.78 | <0.001 |
| Relationship status |  |  |  |  |  |  |  |  |  |  |  |  |  |  |  |
| Partnered | Ref. | - | - | Ref. | - | - | Ref. | - | - | Ref. | - | - | Ref. | - | - |
| Unpartnered | 1.5 | 1.36 - 1.66 | <0.001 | 2.09 | 1.90 - 2.29 | <0.001 | 1.08 | 0.93 - 1.25 | 0.29 | 1.27 | 1.05 - 1.52 | 0.01 | 0.96 | 0.87 - 1.07 | 0.48 |
| Work status |  |  |  |  |  |  |  |  |  |  |  |  |  |  |  |
| Full-time | Ref. | - | - | Ref. | - | - | Ref. | - | - | Ref. | - | - | Ref. | - | - |
| None | 1.71 | 1.46 - 1.99 | <0.001 | 1.40 | 1.21 - 1.61 | <0.001 | 1.19 | 0.95 - 1.48 | 0.12 | 1.19 | 0.88 - 1.60 | 0.25 | 1.31 | 1.11 - 1.54 | <0.001 |
| Part-time | 1.27 | 1.14 - 1.42 | <0.001 | 1.29 | 1.16 - 1.42 | <0.001 | 1.03 | 0.88 - 1.20 | 0.72 | 0.89 | 0.72 - 1.10 | 0.29 | 0.99 | 0.88 - 1.12 | 0.88 |
| Income management |  |  |  |  |  |  |  |  |  |  |  |  |  |  |  |
| Not too bad/easy | Ref. | - | - | Ref. | - | - | Ref. | - | - | Ref. | - | - | Ref. | - | - |
| Difficult sometimes | 0.87 | 0.79 - 0.97 | 0.01 | 0.85 | 0.77 - 0.93 | <0.001 | 0.98 | 0.85 - 1.14 | 0.82 | 1.18 | 0.96 - 1.44 | 0.11 | 0.96 | 0.86 - 1.08 | 0.53 |
| Impossible/difficult always | 0.83 | 0.73 - 0.95 | 0.01 | 0.83 | 0.74 - 0.93 | <0.001 | 1.08 | 0.90 - 1.31 | 0.40 | 1.08 | 0.83 - 1.40 | 0.58 | 1.00 | 0.87 - 1.15 | 0.98 |
| Health care card status |  |  |  |  |  |  |  |  |  |  |  |  |  |  |  |
| No | Ref. | - | - | Ref. | - | - | Ref. | - | - | Ref. | - | - | Ref. | - | - |
| Yes | 0.94 | 0.85 - 1.05 | 0.29 | 0.94 | 0.85 - 1.03 | 0.19 | 1.22 | 1.04 - 1.43 | 0.01 | 1.01 | 0.80 - 1.26 | 0.96 | 1.01 | 0.90 - 1.14 | 0.87 |
| Smoking status |  |  |  |  |  |  |  |  |  |  |  |  |  |  |  |
| Non-smoker | Ref. | - | - | Ref. | - | - | Ref. | - | - | Ref. | - | - | Ref. | - | - |
| Current smoker | 1.28 | 1.12 - 1.46 | <0.001 | 0.65 | 0.57 - 0.73 | <0.001 | 1.53 | 1.26 - 1.85 | <0.001 | 1.31 | 1.03 - 1.66 | 0.03 | 1.87 | 1.64 - 2.13 | <0.001 |
| Alcohol consumption |  |  |  |  |  |  |  |  |  |  |  |  |  |  |  |
| Non-drinker | Ref. | - | - | Ref. | - | - | Ref. | - | - | Ref. | - | - | Ref. | - | - |
| Low risk drinker | 0.49 | 0.40 - 0.60 | <0.001 | 0.64 | 0.52 - 0.78 | <0.001 | 0.82 | 0.60 - 1.11 | 0.20 | 0.63 | 0.44 - 0.92 | 0.02 | 0.44 | 0.36 - 0.55 | <0.001 |
| Infrequent drinker | 0.59 | 0.48 - 0.73 | <0.001 | 0.72 | 0.59 - 0.88 | <0.001 | 0.80 | 0.59 - 1.09 | 0.16 | 0.65 | 0.44 - 0.95 | 0.03 | 0.63 | 0.51 - 0.79 | <0.001 |
| Risky/high risk drinker | 0.59 | 0.44 - 0.81 | <0.001 | 0.49 | 0.36 - 0.66 | <0.001 | 0.77 | 0.49 - 1.22 | 0.27 | 0.85 | 0.48 - 1.50 | 0.57 | 0.56 | 0.40 - 0.77 | <0.001 |
| Body Mass Index |  |  |  |  |  |  |  |  |  |  |  |  |  |  |  |
| Healthy weight | Ref. | - | - | Ref. | - | - | Ref. | - | - | Ref. | - | - | Ref. | - | - |
| Underweight | 1.08 | 0.89 - 1.30 | 0.44 | 0.89 | 0.75 - 1.06 | 0.18 | 0.83 | 0.61 - 1.12 | 0.23 | 0.74 | 0.47 - 1.17 | 0.19 | 1.16 | 0.95 - 1.42 | 0.14 |
| Overweight | 1.14 | 1.01 - 1.28 | 0.03 | 1.34 | 1.20 - 1.48 | <0.001 | 1.35 | 1.14 - 1.60 | <0.001 | 1.37 | 1.10 - 1.70 | <0.001 | 1.21 | 1.06 - 1.37 | <0.001 |
| Obese | 1.65 | 1.42 - 1.91 | <0.001 | 1.47 | 1.28 - 1.68 | <0.001 | 2.31 | 1.86 - 2.87 | <0.001 | 1.70 | 1.31 - 2.20 | <0.001 | 1.59 | 1.37 - 1.85 | <0.001 |
| Psychological distress (K10) |  |  |  |  |  |  |  |  |  |  |  |  |  |  |  |
| Low | Ref. | - | - | Ref. | - | - | Ref. | - | - | Ref. | - | - | Ref. | - | - |
| Moderate | 1.04 | 0.93 - 1.18 | 0.48 | 1.06 | 0.96 - 1.18 | 0.26 | 1.02 | 0.86 - 1.21 | 0.84 | 1.08 | 0.86 - 1.36 | 0.51 | 1.03 | 0.90 - 1.18 | 0.70 |
| High | 0.99 | 0.87 - 1.12 | 0.85 | 0.93 | 0.83 - 1.05 | 0.23 | 0.95 | 0.79 - 1.14 | 0.58 | 0.95 | 0.75 - 1.22 | 0.71 | 1.06 | 0.92 - 1.22 | 0.42 |
| Very high | 1.12 | 0.97 - 1.31 | 0.12 | 1.03 | 0.90 - 1.18 | 0.66 | 1.18 | 0.95 - 1.47 | 0.14 | 1.03 | 0.76 - 1.38 | 0.86 | 1.28 | 1.09 - 1.50 | <0.001 |
| Pregnancy history |  |  |  |  |  |  |  |  |  |  |  |  |  |  |  |
| No | Ref. | - | - | Ref. | - | - | Ref. | - | - | Ref. | - | - | Ref. | - | - |
| Yes | 3.76 | 2.94 - 4.80 | <0.001 | 1.00 | 0.76 - 1.32 | 1.00 | 5.84 | 4.18 - 8.17 | <0.001 | 4.05 | 2.77 - 5.94 | <0.01 | 5.41 | 4.27 - 6.87 | <0.001 |
| History of terminations |  |  |  |  |  |  |  |  |  |  |  |  |  |  |  |
| No | Ref. | - | - | Ref. | - | - | Ref. | - | - | Ref. | - | - | Ref. | - | - |
| Yes | 0.49 | 0.38 - 0.65 | <0.001 | 0.66 | 0.48 - 0.91 | 0.01 | 0.82 | 0.56 - 1.21 | 0.32 | 0.83 | 0.55 - 1.24 | 0.35 | 0.51 | 0.40 - 0.66 | <0.001 |
| History of miscarriage |  |  |  |  |  |  |  |  |  |  |  |  |  |  |  |
| No | Ref. | - | - | Ref. | - | - | Ref. | - | - | Ref. | - | - | Ref. | - | - |
| Yes | 0.64 | 0.48 - 0.85 | <0.001 | 1.21 | 0.88 - 1.67 | 0.25 | 0.51 | 0.34 - 0.76 | <0.001 | 0.97 | 0.64 - 1.47 | 0.89 | 0.92 | 0.71 - 1.20 | 0.55 |
| Menstrual symptoms |  |  |  |  |  |  |  |  |  |  |  |  |  |  |  |
| No | Ref. | - | - | Ref. | - | - | Ref. | - | - | Ref. | - | - | Ref. | - | - |
| Yes | 3.12 | 2.81 - 3.47 | <0.001 | 1.33 | 1.20 - 1.46 | <0.001 | 3.61 | 3.12 - 4.17 | <0.001 | 2.54 | 2.09 - 3.08 | <0.001 | 2.92 | 2.61 - 3.27 | <0.001 |
| History of PCOS |  |  |  |  |  |  |  |  |  |  |  |  |  |  |  |
| No | Ref. | - | - | Ref. | - | - | Ref. | - | - | Ref. | - | - | Ref. | - | - |
| Yes | 0.87 | 0.69 - 1.09 | 0.21 | 0.97 | 0.79 - 1.19 | 0.76 | 0.52 | 0.36 - 0.73 | <0.001 | 0.73 | 0.50 - 1.06 | 0.10 | 1.11 | 0.89 - 1.39 | 0.34 |
| History of endometriosis |  |  |  |  |  |  |  |  |  |  |  |  |  |  |  |
| No | Ref. | - | - | Ref. | - | - | Ref. | - | - | Ref. | - | - | Ref. | - | - |
| Yes | 0.68 | 0.51 - 0.92 | 0.01 | 1.34 | 1.03 - 1.73 | 0.03 | 2.25 | 1.47 - 3.44 | <0.001 | 1.19 | 0.76 - 1.86 | 0.45 | 0.78 | 0.58 - 1.05 | 0.10 |
| Time |  |  |  |  |  |  |  |  |  |  |  |  |  |  |  |
| Survey 1 | Ref. | - | - | Ref. | - | - | Ref. | - | - | Ref. | - | - | Ref. | - | - |
| Survey 3 | 0.98 | 0.88 - 1.10 | 0.79 | 0.94 | 0.85 - 1.04 | 0.23 | 1.81 | 1.54 - 2.12 | <0.001 | 6.46 | 4.86 - 8.60 | <0.001 | 0.76 | 0.67 - 0.87 | <0.001 |
| Survey 5 | 1.54 | 1.32 - 1.81 | <0.001 | 0.87 | 0.75 - 1.01 | 0.07 | 4.27 | 3.37 - 5.40 | <0.001 | 9.60 | 6.80 - 13.55 | <0.001 | 1.04 | 0.87 - 1.24 | 0.68 |

Notes: ESB = English-speaking background; Reference status = Status 4 (pill); A LARC here refers to the use of hormonal long-acting reversible contraception (progestogen-only implant and the progestogen

IUD). Parity was not included in the models due to overlap with pregnancy history.

**Table S6. Full multinomial mixed-effect model for factors associated with contraceptive use by diabetes status for the ALSWH 1989-95 cohort.**

|  | **Condom** | | | **Pill & condom** | | | **LARC & condom** | | | **Other & condom** | | | **None** | | |
| --- | --- | --- | --- | --- | --- | --- | --- | --- | --- | --- | --- | --- | --- | --- | --- |
| **Factor** | **OR** | **95% CI** | ***P*** | **OR** | **95% CI** | ***P*** | **OR** | **95% CI** | ***P*** | **OR** | **95% CI** | ***P*** | **OR** | **95% CI** | ***P*** |
| Diabetes |  |  |  |  |  |  |  |  |  |  |  |  |  |  |  |
| No | Ref. | - | - | Ref. | - | - | Ref. | - | - | Ref. | - | - | Ref. | - | - |
| Yes | 0.86 | 0.66 - 1.12 | 0.27 | 1.10 | 0.73 - 1.67 | 0.65 | 1.06 | 0.80 - 1.41 | 0.67 | 1.18 | 0.78 - 1.79 | 0.42 | 0.95 | 0.75 - 1.21 | 0.69 |
| Age (years) | 0.99 | 0.96 - 1.02 | 0.44 | 0.86 | 0.82 - 0.90 | <0.001 | 0.94 | 0.91 - 0.97 | <0.001 | 1.07 | 1.01 - 1.13 | 0.01 | 1.05 | 1.02 - 1.09 | <0.001 |
| Country of birth |  |  |  |  |  |  |  |  |  |  |  |  |  |  |  |
| Australia | Ref. | - | - | Ref. | - | - | Ref. | - | - | Ref. | - | - | Ref. | - | - |
| Other ESB | 0.99 | 0.78 - 1.27 | 0.96 | 0.85 | 0.57 - 1.28 | 0.44 | 1.24 | 0.95 - 1.62 | 0.11 | 1.24 | 0.84 - 1.83 | 0.29 | 0.86 | 0.68 - 1.09 | 0.22 |
| Non-ESB | 1.66 | 1.32 - 2.09 | <0.001 | 0.62 | 0.42 - 0.93 | 0.02 | 0.93 | 0.70 - 1.24 | 0.62 | 1.47 | 0.98 - 2.21 | 0.07 | 1.56 | 1.25 - 1.94 | <0.001 |
| Area of residence |  |  |  |  |  |  |  |  |  |  |  |  |  |  |  |
| Major cities | Ref. | - | - | Ref. | - | - | Ref. | - | - | Ref. | - | - | Ref. | - | - |
| Inner regional | 0.94 | 0.83 - 1.06 | 0.33 | 1.17 | 0.97 - 1.40 | 0.09 | 1.10 | 0.97 - 1.26 | 0.15 | 0.87 | 0.68 - 1.10 | 0.24 | 0.94 | 0.84 - 1.06 | 0.30 |
| Outer regional/remote/very remote | 1.04 | 0.88 - 1.23 | 0.64 | 1.25 | 0.98 - 1.61 | 0.07 | 1.44 | 1.21 - 1.72 | <0.001 | 1.38 | 1.04 - 1.84 | 0.03 | 1.07 | 0.91 - 1.25 | 0.44 |
| Education |  |  |  |  |  |  |  |  |  |  |  |  |  |  |  |
| Year 12 or below | Ref. | - | - | Ref. | - | - | Ref. | - | - | Ref. | - | - | Ref. | - | - |
| Certificate/diploma | 0.96 | 0.85 - 1.07 | 0.43 | 0.91 | 0.77 - 1.08 | 0.28 | 1.11 | 0.98 - 1.26 | 0.09 | 0.97 | 0.78 - 1.21 | 0.77 | 1.07 | 0.96 - 1.19 | 0.24 |
| University | 0.94 | 0.84 - 1.06 | 0.30 | 1.16 | 0.98 - 1.37 | 0.09 | 1.09 | 0.96 - 1.24 | 0.18 | 0.72 | 0.57 - 0.90 | 0.01 | 0.68 | 0.60 - 0.77 | <0.001 |
| Relationship status |  |  |  |  |  |  |  |  |  |  |  |  |  |  |  |
| Partnered | Ref. | - | - | Ref. | - | - | Ref. | - | - | Ref. | - | - | Ref. | - | - |
| Unpartnered | 1.63 | 1.48 - 1.79 | <0.001 | 2.94 | 2.55 - 3.40 | <0.001 | 1.28 | 1.16 - 1.42 | <0.001 | 1.32 | 1.11 - 1.57 | <0.001 | 0.96 | 0.87 - 1.05 | 0.34 |
| Work status |  |  |  |  |  |  |  |  |  |  |  |  |  |  |  |
| Full-time | Ref. | - | - | Ref. | - | - | Ref. | - | - | Ref. | - | - | Ref. | - | - |
| None | 1.73 | 1.49 - 2.00 | <0.001 | 1.43 | 1.16 - 1.77 | <0.001 | 1.23 | 1.05 - 1.44 | 0.01 | 1.19 | 0.90 - 1.58 | 0.23 | 1.27 | 1.10 - 1.47 | <0.001 |
| Part-time | 1.32 | 1.19 - 1.46 | <0.001 | 1.43 | 1.24 - 1.66 | <0.001 | 1.07 | 0.96 - 1.20 | 0.21 | 0.91 | 0.74 - 1.11 | 0.34 | 0.99 | 0.89 - 1.10 | 0.81 |
| Income management |  |  |  |  |  |  |  |  |  |  |  |  |  |  |  |
| Not too bad/easy | Ref. | - | - | Ref. | - | - | Ref. | - | - | Ref. | - | - | Ref. | - | - |
| Difficult sometimes | 0.85 | 0.77 - 0.94 | <0.001 | 0.78 | 0.68 - 0.90 | <0.001 | 0.91 | 0.82 - 1.01 | 0.09 | 1.15 | 0.95 - 1.39 | 0.17 | 0.96 | 0.87 - 1.06 | 0.43 |
| Impossible/difficult always | 0.8 | 0.70 - 0.90 | <0.001 | 0.78 | 0.66 - 0.93 | 0.01 | 0.94 | 0.82 - 1.07 | 0.35 | 1.01 | 0.78 - 1.30 | 0.93 | 0.98 | 0.86 - 1.10 | 0.70 |
| Health care card status |  |  |  |  |  |  |  |  |  |  |  |  |  |  |  |
| No | Ref. | - | - | Ref. | - | - | Ref. | - | - | Ref. | - | - | Ref. | - | - |
| Yes | 0.95 | 0.86 - 1.05 | 0.33 | 0.96 | 0.83 - 1.11 | 0.57 | 1.19 | 1.06 - 1.33 | <0.001 | 1.02 | 0.82 - 1.28 | 0.84 | 1.01 | 0.91 - 1.13 | 0.83 |
| Smoking status |  |  |  |  |  |  |  |  |  |  |  |  |  |  |  |
| Non-smoker | Ref. | - | - | Ref. | - | - | Ref. | - | - | Ref. | - | - | Ref. | - | - |
| Current smoker | 1.06 | 0.94 - 1.19 | 0.34 | 0.45 | 0.37 - 0.55 | <0.001 | 1.09 | 0.96 - 1.24 | 0.18 | 1.09 | 0.88 - 1.36 | 0.44 | 1.69 | 1.51 - 1.88 | <0.001 |
| Alcohol consumption |  |  |  |  |  |  |  |  |  |  |  |  |  |  |  |
| Non-drinker | Ref. | - | - | Ref. | - | - | Ref. | - | - | Ref. | - | - | Ref. | - | - |
| Low risk drinker | 0.51 | 0.42 - 0.61 | <0.001 | 0.62 | 0.46 - 0.83 | <0.001 | 0.79 | 0.63 - 0.99 | 0.04 | 0.67 | 0.47 - 0.95 | 0.03 | 0.48 | 0.40 - 0.58 | <0.001 |
| Infrequent drinker | 0.60 | 0.49 - 0.73 | <0.001 | 0.68 | 0.51 - 0.92 | 0.01 | 0.80 | 0.64 - 1.00 | 0.05 | 0.67 | 0.46 - 0.96 | 0.03 | 0.66 | 0.55 - 0.80 | <0.001 |
| Risky/high risk drinker | 0.59 | 0.44 - 0.78 | <0.001 | 0.44 | 0.28 - 0.69 | <0.001 | 0.71 | 0.51 - 0.99 | 0.04 | 0.85 | 0.50 - 1.47 | 0.57 | 0.58 | 0.44 - 0.76 | <0.001 |
| Body Mass Index |  |  |  |  |  |  |  |  |  |  |  |  |  |  |  |
| Healthy weight | Ref. | - | - | Ref. | - | - | Ref. | - | - | Ref. | - | - | Ref. | - | - |
| Underweight | 1.03 | 0.86 - 1.23 | 0.76 | 0.80 | 0.61 - 1.05 | 0.11 | 0.78 | 0.63 - 0.96 | 0.02 | 0.72 | 0.46 - 1.12 | 0.14 | 1.13 | 0.95 - 1.35 | 0.16 |
| Overweight | 1.16 | 1.04 - 1.30 | 0.01 | 1.46 | 1.24 - 1.72 | <0.001 | 1.33 | 1.18 - 1.50 | <0.001 | 1.37 | 1.12 - 1.68 | <0.001 | 1.20 | 1.07 - 1.34 | <0.001 |
| Obese | 1.53 | 1.33 - 1.75 | <0.001 | 1.52 | 1.23 - 1.88 | <0.001 | 1.80 | 1.55 - 2.09 | <0.001 | 1.50 | 1.19 - 1.91 | <0.001 | 1.44 | 1.27 - 1.64 | <0.001 |
| Psychological distress (K10) |  |  |  |  |  |  |  |  |  |  |  |  |  |  |  |
| Low | Ref. | - | - | Ref. | - | - | Ref. | - | - | Ref. | - | - | Ref. | - | - |
| Moderate | 1.05 | 0.93 - 1.17 | 0.44 | 1.11 | 0.95 - 1.30 | 0.20 | 1.02 | 0.90 - 1.15 | 0.76 | 1.08 | 0.87 - 1.34 | 0.50 | 1.01 | 0.90 - 1.15 | 0.84 |
| High | 0.98 | 0.87 - 1.11 | 0.74 | 0.91 | 0.77 - 1.09 | 0.31 | 0.93 | 0.82 - 1.07 | 0.31 | 0.94 | 0.75 - 1.19 | 0.63 | 1.05 | 0.93 - 1.20 | 0.41 |
| Very high | 1.06 | 0.92 - 1.22 | 0.43 | 0.95 | 0.78 - 1.17 | 0.64 | 1.06 | 0.91 - 1.23 | 0.49 | 0.97 | 0.73 - 1.28 | 0.83 | 1.23 | 1.07 - 1.42 | <0.001 |
| Pregnancy history |  |  |  |  |  |  |  |  |  |  |  |  |  |  |  |
| No | Ref. | - | - | Ref. | - | - | Ref. | - | - | Ref. | - | - | Ref. | - | - |
| Yes | 2.71 | 2.18 - 3.37 | <0.001 | 0.67 | 0.46 - 1.00 | 0.05 | 2.77 | 2.19 - 3.51 | <0.001 | 2.79 | 1.98 - 3.93 | <0.01 | 4.09 | 3.40 - 4.93 | <0.001 |
| History of terminations |  |  |  |  |  |  |  |  |  |  |  |  |  |  |  |
| No | Ref. | - | - | Ref. | - | - | Ref. | - | - | Ref. | - | - | Ref. | - | - |
| Yes | 0.51 | 0.40 - 0.66 | <0.001 | 0.73 | 0.47 - 1.14 | 0.16 | 0.82 | 0.63 - 1.06 | 0.13 | 0.84 | 0.58 - 1.22 | 0.36 | 0.53 | 0.43 - 0.65 | <0.001 |
| History of miscarriage |  |  |  |  |  |  |  |  |  |  |  |  |  |  |  |
| No | Ref. | - | - | Ref. | - | - | Ref. | - | - | Ref. | - | - | Ref. | - | - |
| Yes | 0.65 | 0.50 - 0.84 | <0.001 | 1.17 | 0.74 - 1.84 | 0.51 | 0.60 | 0.45 - 0.79 | <0.001 | 1.02 | 0.70 - 1.48 | 0.93 | 0.97 | 0.78 - 1.20 | 0.77 |
| Menstrual symptoms |  |  |  |  |  |  |  |  |  |  |  |  |  |  |  |
| No | Ref. | - | - | Ref. | - | - | Ref. | - | - | Ref. | - | - | Ref. | - | - |
| Yes | 2.72 | 2.47 - 2.99 | <0.001 | 1.28 | 1.11 - 1.47 | <0.001 | 2.58 | 2.32 - 2.86 | <0.001 | 2.08 | 1.73 - 2.49 | <0.001 | 2.46 | 2.23 - 2.70 | <0.001 |
| History of PCOS |  |  |  |  |  |  |  |  |  |  |  |  |  |  |  |
| No | Ref. | - | - | Ref. | - | - | Ref. | - | - | Ref. | - | - | Ref. | - | - |
| Yes | 0.91 | 0.74 - 1.12 | 0.40 | 0.89 | 0.64 - 1.25 | 0.51 | 0.66 | 0.52 - 0.83 | <0.001 | 0.81 | 0.57 - 1.13 | 0.21 | 1.19 | 1.00 - 1.43 | 0.06 |
| History of endometriosis |  |  |  |  |  |  |  |  |  |  |  |  |  |  |  |
| No | Ref. | - | - | Ref. | - | - | Ref. | - | - | Ref. | - | - | Ref. | - | - |
| Yes | 0.73 | 0.55 - 0.96 | 0.03 | 1.52 | 1.00 - 2.33 | 0.05 | 1.90 | 1.44 - 2.51 | <0.001 | 1.22 | 0.81 - 1.83 | 0.34 | 0.80 | 0.63 - 1.03 | 0.08 |
| Time |  |  |  |  |  |  |  |  |  |  |  |  |  |  |  |
| Survey 1 | Ref. | - | - | Ref. | - | - | Ref. | - | - | Ref. | - | - | Ref. | - | - |
| Survey 3 | 0.89 | 0.80 - 0.99 | 0.03 | 0.82 | 0.71 - 0.95 | 0.01 | 1.33 | 1.18 - 1.49 | <0.001 | 5.72 | 4.33 - 7.55 | <0.001 | 0.70 | 0.62 - 0.79 | <0.001 |
| Survey 5 | 1.21 | 1.04 - 1.40 | 0.01 | 0.65 | 0.52 - 0.82 | <0.001 | 2.07 | 1.76 - 2.42 | <0.001 | 7.05 | 5.09 - 9.75 | <0.001 | 0.83 | 0.71 - 0.96 | 0.01 |

Notes: ESB = English-speaking background; Reference status = Status 4 (pill); A LARC here refers to the use of hormonal long-acting reversible contraception (progestogen-only implant and the progestogen

IUD). Parity was not included in the models due to overlap with pregnancy history.

**Table S7. Full multinomial mixed-effect model for factors associated with contraceptive use by asthma status for the ALSWH 1989-95 cohort.**

|  | **Condom** | | | **Pill & condom** | | | **LARC & condom** | | | **Other & condom** | | | **None** | | |
| --- | --- | --- | --- | --- | --- | --- | --- | --- | --- | --- | --- | --- | --- | --- | --- |
| **Factor** | **OR** | **95% CI** | ***P*** | **OR** | **95% CI** | ***P*** | **OR** | **95% CI** | ***P*** | **OR** | **95% CI** | ***P*** | **OR** | **95% CI** | ***P*** |
| Asthma |  |  |  |  |  |  |  |  |  |  |  |  |  |  |  |
| No | Ref. | - | - | Ref. | - | - | Ref. | - | - | Ref. | - | - | Ref. | - | - |
| Yes | 0.89 | 0.78 - 1.02 | 0.09 | 1.15 | 0.93 - 1.41 | 0.20 | 1.04 | 0.90 - 1.20 | 0.58 | 1.22 | 0.98 - 1.51 | 0.07 | 0.88 | 0.78 - 1.00 | 0.05 |
| Age (years) | 0.99 | 0.96 - 1.02 | 0.43 | 0.86 | 0.82 - 0.90 | <0.001 | 0.94 | 0.91 - 0.97 | <0.001 | 1.07 | 1.01 - 1.13 | 0.01 | 1.05 | 1.02 - 1.08 | <0.001 |
| Country of birth |  |  |  |  |  |  |  |  |  |  |  |  |  |  |  |
| Australia | Ref. | - | - | Ref. | - | - | Ref. | - | - | Ref. | - | - | Ref. | - | - |
| Other ESB | 0.99 | 0.78 - 1.26 | 0.94 | 0.86 | 0.57 - 1.28 | 0.46 | 1.24 | 0.96 - 1.62 | 0.10 | 1.25 | 0.84 - 1.85 | 0.27 | 0.86 | 0.68 - 1.09 | 0.20 |
| Non-ESB | 1.66 | 1.32 - 2.08 | <0.001 | 0.62 | 0.42 - 0.93 | 0.02 | 0.93 | 0.70 - 1.24 | 0.63 | 1.47 | 0.98 - 2.22 | 0.06 | 1.56 | 1.25 - 1.94 | <0.001 |
| Area of residence |  |  |  |  |  |  |  |  |  |  |  |  |  |  |  |
| Major cities | Ref. | - | - | Ref. | - | - | Ref. | - | - | Ref. | - | - | Ref. | - | - |
| Inner regional | 0.94 | 0.83 - 1.06 | 0.33 | 1.17 | 0.97 - 1.40 | 0.09 | 1.10 | 0.97 - 1.26 | 0.15 | 0.87 | 0.68 - 1.10 | 0.23 | 0.94 | 0.84 - 1.06 | 0.30 |
| Outer regional/remote/very remote | 1.04 | 0.88 - 1.23 | 0.65 | 1.26 | 0.98 - 1.61 | 0.07 | 1.44 | 1.21 - 1.72 | <0.001 | 1.38 | 1.04 - 1.84 | 0.03 | 1.06 | 0.91 - 1.25 | 0.45 |
| Education |  |  |  |  |  |  |  |  |  |  |  |  |  |  |  |
| Year 12 or below | Ref. | - | - | Ref. | - | - | Ref. | - | - | Ref. | - | - | Ref. | - | - |
| Certificate/diploma | 0.95 | 0.85 - 1.07 | 0.41 | 0.91 | 0.77 - 1.08 | 0.29 | 1.11 | 0.98 - 1.26 | 0.09 | 0.97 | 0.78 - 1.21 | 0.79 | 1.07 | 0.96 - 1.19 | 0.24 |
| University | 0.94 | 0.84 - 1.06 | 0.31 | 1.16 | 0.98 - 1.37 | 0.08 | 1.09 | 0.96 - 1.24 | 0.18 | 0.72 | 0.57 - 0.90 | 0.01 | 0.68 | 0.60 - 0.77 | <0.001 |
| Relationship status |  |  |  |  |  |  |  |  |  |  |  |  |  |  |  |
| Partnered | Ref. | - | - | Ref. | - | - | Ref. | - | - | Ref. | - | - | Ref. | - | - |
| Unpartnered | 1.63 | 1.48 - 1.79 | <0.001 | 2.95 | 2.55 - 3.40 | <0.001 | 1.28 | 1.15 - 1.42 | <0.001 | 1.32 | 1.11 - 1.58 | <0.001 | 0.95 | 0.87 - 1.05 | 0.32 |
| Work status |  |  |  |  |  |  |  |  |  |  |  |  |  |  |  |
| Full-time | Ref. | - | - | Ref. | - | - | Ref. | - | - | Ref. | - | - | Ref. | - | - |
| None | 1.73 | 1.49 - 2.00 | <0.001 | 1.43 | 1.16 - 1.77 | <0.001 | 1.23 | 1.05 - 1.44 | 0.01 | 1.19 | 0.90 - 1.58 | 0.23 | 1.27 | 1.10 - 1.47 | <0.001 |
| Part-time | 1.31 | 1.18 - 1.46 | <0.001 | 1.43 | 1.24 - 1.66 | <0.001 | 1.07 | 0.96 - 1.20 | 0.21 | 0.91 | 0.75 - 1.11 | 0.36 | 0.99 | 0.89 - 1.10 | 0.78 |
| Income management |  |  |  |  |  |  |  |  |  |  |  |  |  |  |  |
| Not too bad/easy | Ref. | - | - | Ref. | - | - | Ref. | - | - | Ref. | - | - | Ref. | - | - |
| Difficult sometimes | 0.85 | 0.77 - 0.94 | <0.001 | 0.78 | 0.68 - 0.90 | <0.001 | 0.91 | 0.82 - 1.01 | 0.09 | 1.14 | 0.94 - 1.39 | 0.17 | 0.96 | 0.87 - 1.06 | 0.44 |
| Impossible/difficult always | 0.80 | 0.70 - 0.90 | <0.001 | 0.78 | 0.66 - 0.93 | 0.01 | 0.94 | 0.82 - 1.07 | 0.35 | 1.01 | 0.78 - 1.30 | 0.96 | 0.98 | 0.87 - 1.11 | 0.73 |
| Health care card status |  |  |  |  |  |  |  |  |  |  |  |  |  |  |  |
| No | Ref. | - | - | Ref. | - | - | Ref. | - | - | Ref. | - | - | Ref. | - | - |
| Yes | 0.95 | 0.86 - 1.06 | 0.35 | 0.96 | 0.83 - 1.11 | 0.55 | 1.19 | 1.06 - 1.33 | <0.001 | 1.02 | 0.82 - 1.27 | 0.85 | 1.01 | 0.91 - 1.13 | 0.80 |
| Smoking status |  |  |  |  |  |  |  |  |  |  |  |  |  |  |  |
| Non-smoker | Ref. | - | - | Ref. | - | - | Ref. | - | - | Ref. | - | - | Ref. | - | - |
| Current smoker | 1.06 | 0.94 - 1.19 | 0.34 | 0.45 | 0.37 - 0.55 | <0.001 | 1.09 | 0.96 - 1.24 | 0.18 | 1.09 | 0.87 - 1.36 | 0.44 | 1.69 | 1.52 - 1.88 | <0.001 |
| Alcohol consumption |  |  |  |  |  |  |  |  |  |  |  |  |  |  |  |
| Non-drinker | Ref. | - | - | Ref. | - | - | Ref. | - | - | Ref. | - | - | Ref. | - | - |
| Low risk drinker | 0.51 | 0.42 - 0.61 | <0.001 | 0.62 | 0.46 - 0.83 | <0.001 | 0.79 | 0.63 - 0.99 | 0.04 | 0.67 | 0.47 - 0.95 | 0.03 | 0.48 | 0.40 - 0.57 | <0.001 |
| Infrequent drinker | 0.60 | 0.49 - 0.73 | <0.001 | 0.68 | 0.51 - 0.92 | 0.01 | 0.80 | 0.64 - 1.00 | 0.05 | 0.67 | 0.46 - 0.95 | 0.03 | 0.66 | 0.55 - 0.80 | <0.001 |
| Risky/high risk drinker | 0.59 | 0.44 - 0.78 | <0.001 | 0.44 | 0.28 - 0.69 | <0.001 | 0.71 | 0.51 - 0.99 | 0.04 | 0.85 | 0.49 - 1.46 | 0.56 | 0.58 | 0.44 - 0.76 | <0.001 |
| Body Mass Index |  |  |  |  |  |  |  |  |  |  |  |  |  |  |  |
| Healthy weight | Ref. | - | - | Ref. | - | - | Ref. | - | - | Ref. | - | - | Ref. | - | - |
| Underweight | 1.03 | 0.86 - 1.22 | 0.77 | 0.81 | 0.61 - 1.05 | 0.12 | 0.78 | 0.63 - 0.96 | 0.02 | 0.72 | 0.46 - 1.12 | 0.14 | 1.13 | 0.95 - 1.35 | 0.17 |
| Overweight | 1.16 | 1.04 - 1.30 | 0.01 | 1.46 | 1.24 - 1.71 | <0.001 | 1.33 | 1.18 - 1.50 | <0.001 | 1.37 | 1.11 - 1.67 | <0.001 | 1.20 | 1.08 - 1.34 | <0.001 |
| Obese | 1.53 | 1.34 - 1.76 | <0.001 | 1.51 | 1.22 - 1.87 | <0.001 | 1.80 | 1.55 - 2.08 | <0.001 | 1.49 | 1.18 - 1.89 | <0.001 | 1.45 | 1.28 - 1.65 | <0.001 |
| Psychological distress (K10) |  |  |  |  |  |  |  |  |  |  |  |  |  |  |  |
| Low | Ref. | - | - | Ref. | - | - | Ref. | - | - | Ref. | - | - | Ref. | - | - |
| Moderate | 1.05 | 0.93 - 1.17 | 0.42 | 1.11 | 0.95 - 1.30 | 0.21 | 1.02 | 0.90 - 1.15 | 0.76 | 1.08 | 0.86 - 1.34 | 0.52 | 1.02 | 0.90 - 1.15 | 0.81 |
| High | 0.98 | 0.87 - 1.11 | 0.78 | 0.91 | 0.77 - 1.09 | 0.30 | 0.93 | 0.82 - 1.07 | 0.31 | 0.94 | 0.74 - 1.19 | 0.61 | 1.06 | 0.93 - 1.20 | 0.38 |
| Very high | 1.06 | 0.92 - 1.22 | 0.39 | 0.95 | 0.77 - 1.17 | 0.61 | 1.06 | 0.90 - 1.23 | 0.49 | 0.96 | 0.73 - 1.27 | 0.79 | 1.24 | 1.08 - 1.43 | <0.001 |
| Pregnancy history |  |  |  |  |  |  |  |  |  |  |  |  |  |  |  |
| No | Ref. | - | - | Ref. | - | - | Ref. | - | - | Ref. | - | - | Ref. | - | - |
| Yes | 2.70 | 2.17 - 3.36 | <0.001 | 0.68 | 0.46 - 1.00 | 0.05 | 2.77 | 2.19 - 3.51 | <0.001 | 2.80 | 1.99 - 3.95 | <0.001 | 4.08 | 3.39 - 4.91 | <0.001 |
| History of terminations |  |  |  |  |  |  |  |  |  |  |  |  |  |  |  |
| No | Ref. | - | - | Ref. | - | - | Ref. | - | - | Ref. | - | - | Ref. | - | - |
| Yes | 0.52 | 0.40 - 0.66 | <0.001 | 0.73 | 0.46 - 1.13 | 0.16 | 0.82 | 0.63 - 1.06 | 0.13 | 0.84 | 0.58 - 1.21 | 0.34 | 0.53 | 0.43 - 0.66 | <0.001 |
| History of miscarriage |  |  |  |  |  |  |  |  |  |  |  |  |  |  |  |
| No | Ref. | - | - | Ref. | - | - | Ref. | - | - | Ref. | - | - | Ref. | - | - |
| Yes | 0.65 | 0.50 - 0.84 | <0.001 | 1.16 | 0.73 - 1.84 | 0.52 | 0.59 | 0.45 - 0.78 | <0.001 | 1.01 | 0.70 - 1.48 | 0.94 | 0.97 | 0.79 - 1.20 | 0.79 |
| Menstrual symptoms |  |  |  |  |  |  |  |  |  |  |  |  |  |  |  |
| No | Ref. | - | - | Ref. | - | - | Ref. | - | - | Ref. | - | - | Ref. | - | - |
| Yes | 2.72 | 2.47 - 2.99 | <0.001 | 1.28 | 1.11 - 1.47 | <0.001 | 2.58 | 2.32 - 2.86 | <0.001 | 2.07 | 1.73 - 2.48 | <0.001 | 2.46 | 2.24 - 2.70 | <0.001 |
| History of PCOS |  |  |  |  |  |  |  |  |  |  |  |  |  |  |  |
| No | Ref. | - | - | Ref. | - | - | Ref. | - | - | Ref. | - | - | Ref. | - | - |
| Yes | 0.91 | 0.74 - 1.12 | 0.37 | 0.89 | 0.64 - 1.25 | 0.51 | 0.66 | 0.52 - 0.83 | <0.001 | 0.81 | 0.57 - 1.13 | 0.22 | 1.19 | 1.00 - 1.43 | 0.05 |
| History of endometriosis |  |  |  |  |  |  |  |  |  |  |  |  |  |  |  |
| No | Ref. | - | - | Ref. | - | - | Ref. | - | - | Ref. | - | - | Ref. | - | - |
| Yes | 0.73 | 0.55 - 0.97 | 0.03 | 1.51 | 0.99 - 2.30 | 0.06 | 1.90 | 1.44 - 2.50 | <0.001 | 1.21 | 0.81 - 1.81 | 0.36 | 0.80 | 0.63 - 1.03 | 0.08 |
| Time |  |  |  |  |  |  |  |  |  |  |  |  |  |  |  |
| Survey 1 | Ref. | - | - | Ref. | - | - | Ref. | - | - | Ref. | - | - | Ref. | - | - |
| Survey 3 | 0.89 | 0.80 - 0.99 | 0.03 | 0.82 | 0.71 - 0.95 | 0.01 | 1.33 | 1.18 - 1.49 | <0.001 | 5.70 | 4.32 - 7.52 | <0.001 | 0.70 | 0.63 - 0.79 | <0.001 |
| Survey 5 | 1.21 | 1.05 - 1.40 | 0.01 | 0.65 | 0.52 - 0.82 | <0.001 | 2.06 | 1.76 - 2.42 | <0.001 | 7.02 | 5.08 - 9.71 | <0.001 | 0.83 | 0.72 - 0.96 | 0.01 |

Notes: ESB = English-speaking background; Reference status = Status 4 (pill); A LARC here refers to the use of hormonal long-acting reversible contraception (progestogen-only implant and the progestogen

IUD). Parity was not included in the models due to overlap with pregnancy history.

**Table S8. Full multinomial mixed-effect model for factors associated with contraceptive use by autoinflammatory disease status for the ALSWH 1989-95 cohort.**

|  | **Condom** | | | **Pill & condom** | | | **LARC & condom** | | | **Other & condom** | | | **None** | | |
| --- | --- | --- | --- | --- | --- | --- | --- | --- | --- | --- | --- | --- | --- | --- | --- |
| **Factor** | **OR** | **95% CI** | ***P*** | **OR** | **95% CI** | ***P*** | **OR** | **95% CI** | ***P*** | **OR** | **95% CI** | ***P*** | **OR** | **95% CI** | ***P*** |
| Autoinflammatory disease |  |  |  |  |  |  |  |  |  |  |  |  |  |  |  |
| No | Ref. | - | - | Ref. | - | - | Ref. | - | - | Ref. | - | - | Ref. | - | - |
| Yes | 0.90 | 0.67 - 1.19 | 0.46 | 1.38 | 1.09 - 1.75 | 0.01 | 1.58 | 1.04 - 2.41 | 0.03 | 1.69 | 1.11 - 2.57 | 0.01 | 1.03 | 0.77 - 1.38 | 0.85 |
| Age (years) | 1.01 | 0.97 - 1.04 | 0.72 | 0.92 | 0.90 - 0.95 | <0.001 | 0.94 | 0.89 - 0.99 | 0.01 | 1.07 | 1.01 - 1.14 | 0.02 | 1.06 | 1.02 - 1.09 | <0.001 |
| Country of birth |  |  |  |  |  |  |  |  |  |  |  |  |  |  |  |
| Australia | Ref. | - | - | Ref. | - | - | Ref. | - | - | Ref. | - | - | Ref. | - | - |
| Other ESB | 1.04 | 0.80 - 1.35 | 0.77 | 0.96 | 0.76 - 1.21 | 0.72 | 1.41 | 0.93 - 2.15 | 0.11 | 1.33 | 0.86 - 2.05 | 0.20 | 0.91 | 0.69 - 1.21 | 0.53 |
| Non-ESB | 1.87 | 1.45 - 2.41 | <0.001 | 0.81 | 0.63 - 1.03 | 0.09 | 1.09 | 0.71 - 1.68 | 0.70 | 1.66 | 1.06 - 2.60 | 0.03 | 1.70 | 1.29 - 2.22 | <0.001 |
| Area of residence |  |  |  |  |  |  |  |  |  |  |  |  |  |  |  |
| Major cities | Ref. | - | - | Ref. | - | - | Ref. | - | - | Ref. | - | - | Ref. | - | - |
| Inner regional | 0.93 | 0.82 - 1.06 | 0.28 | 1.08 | 0.96 - 1.21 | 0.20 | 1.14 | 0.94 - 1.38 | 0.18 | 0.87 | 0.68 - 1.12 | 0.28 | 0.94 | 0.82 - 1.08 | 0.38 |
| Outer regional/remote/very remote | 1.05 | 0.88 - 1.26 | 0.59 | 1.23 | 1.05 - 1.44 | 0.01 | 1.55 | 1.20 - 2.01 | <0.001 | 1.44 | 1.06 - 1.95 | 0.02 | 1.10 | 0.91 - 1.33 | 0.31 |
| Education |  |  |  |  |  |  |  |  |  |  |  |  |  |  |  |
| Year 12 or below | Ref. | - | - | Ref. | - | - | Ref. | - | - | Ref. | - | - | Ref. | - | - |
| Certificate/diploma | 1.00 | 0.89 - 1.13 | 0.94 | 0.98 | 0.88 - 1.10 | 0.79 | 1.24 | 1.03 - 1.48 | 0.02 | 1.01 | 0.80 - 1.27 | 0.93 | 1.09 | 0.96 - 1.23 | 0.17 |
| University | 0.95 | 0.84 - 1.08 | 0.46 | 1.14 | 1.02 - 1.27 | 0.02 | 1.20 | 1.00 - 1.44 | 0.05 | 0.73 | 0.57 - 0.93 | 0.01 | 0.68 | 0.59 - 0.78 | <0.001 |
| Relationship status |  |  |  |  |  |  |  |  |  |  |  |  |  |  |  |
| Partnered | Ref. | - | - | Ref. | - | - | Ref. | - | - | Ref. | - | - | Ref. | - | - |
| Unpartnered | 1.50 | 1.36 - 1.66 | <0.001 | 2.09 | 1.90 - 2.29 | <0.001 | 1.08 | 0.94 - 1.25 | 0.29 | 1.27 | 1.05 - 1.52 | 0.01 | 0.96 | 0.87 - 1.07 | 0.47 |
| Work status |  |  |  |  |  |  |  |  |  |  |  |  |  |  |  |
| Full-time | Ref. | - | - | Ref. | - | - | Ref. | - | - | Ref. | - | - | Ref. | - | - |
| None | 1.71 | 1.47 - 1.99 | <0.001 | 1.40 | 1.21 - 1.61 | <0.001 | 1.18 | 0.95 - 1.48 | 0.13 | 1.19 | 0.88 - 1.59 | 0.26 | 1.31 | 1.11 - 1.54 | <0.001 |
| Part-time | 1.27 | 1.14 - 1.41 | <0.001 | 1.28 | 1.16 - 1.42 | <0.001 | 1.03 | 0.88 - 1.20 | 0.76 | 0.89 | 0.72 - 1.09 | 0.27 | 0.99 | 0.88 - 1.11 | 0.86 |
| Income management |  |  |  |  |  |  |  |  |  |  |  |  |  |  |  |
| Not too bad/easy | Ref. | - | - | Ref. | - | - | Ref. | - | - | Ref. | - | - | Ref. | - | - |
| Difficult sometimes | 0.87 | 0.79 - 0.97 | 0.01 | 0.85 | 0.77 - 0.93 | <0.001 | 0.98 | 0.85 - 1.14 | 0.81 | 1.17 | 0.96 - 1.43 | 0.12 | 0.96 | 0.86 - 1.08 | 0.52 |
| Impossible/difficult always | 0.83 | 0.73 - 0.95 | 0.01 | 0.83 | 0.74 - 0.93 | <0.001 | 1.08 | 0.90 - 1.31 | 0.40 | 1.08 | 0.83 - 1.41 | 0.57 | 1.00 | 0.87 - 1.15 | 0.99 |
| Health care card status |  |  |  |  |  |  |  |  |  |  |  |  |  |  |  |
| No | Ref. | - | - | Ref. | - | - | Ref. | - | - | Ref. | - | - | Ref. | - | - |
| Yes | 0.95 | 0.85 - 1.05 | 0.32 | 0.94 | 0.85 - 1.03 | 0.19 | 1.22 | 1.04 - 1.43 | 0.01 | 1.01 | 0.80 - 1.27 | 0.95 | 1.01 | 0.90 - 1.14 | 0.83 |
| Smoking status |  |  |  |  |  |  |  |  |  |  |  |  |  |  |  |
| Non-smoker | Ref. | - | - | Ref. | - | - | Ref. | - | - | Ref. | - | - | Ref. | - | - |
| Current smoker | 1.28 | 1.13 - 1.46 | <0.001 | 0.65 | 0.57 - 0.73 | <0.001 | 1.53 | 1.27 - 1.86 | <0.001 | 1.32 | 1.04 - 1.67 | 0.02 | 1.87 | 1.64 - 2.13 | <0.001 |
| Alcohol consumption |  |  |  |  |  |  |  |  |  |  |  |  |  |  |  |
| Non-drinker | Ref. | - | - | Ref. | - | - | Ref. | - | - | Ref. | - | - | Ref. | - | - |
| Low risk drinker | 0.48 | 0.39 - 0.60 | <0.001 | 0.64 | 0.53 - 0.78 | <0.001 | 0.82 | 0.60 - 1.12 | 0.20 | 0.64 | 0.44 - 0.93 | 0.02 | 0.44 | 0.36 - 0.55 | <0.001 |
| Infrequent drinker | 0.59 | 0.48 - 0.73 | <0.001 | 0.72 | 0.59 - 0.88 | <0.001 | 0.80 | 0.59 - 1.09 | 0.17 | 0.66 | 0.45 - 0.96 | 0.03 | 0.63 | 0.51 - 0.79 | <0.001 |
| Risky/high risk drinker | 0.59 | 0.43 - 0.80 | <0.001 | 0.49 | 0.36 - 0.67 | <0.001 | 0.77 | 0.49 - 1.23 | 0.28 | 0.86 | 0.48 - 1.52 | 0.60 | 0.56 | 0.40 - 0.77 | <0.001 |
| Body Mass Index |  |  |  |  |  |  |  |  |  |  |  |  |  |  |  |
| Healthy weight | Ref. | - | - | Ref. | - | - | Ref. | - | - | Ref. | - | - | Ref. | - | - |
| Underweight | 1.08 | 0.89 - 1.30 | 0.44 | 0.89 | 0.75 - 1.06 | 0.19 | 0.83 | 0.62 - 1.13 | 0.24 | 0.75 | 0.47 - 1.18 | 0.21 | 1.17 | 0.96 - 1.43 | 0.13 |
| Overweight | 1.14 | 1.01 - 1.28 | 0.03 | 1.34 | 1.21 - 1.48 | <0.001 | 1.35 | 1.14 - 1.60 | <0.001 | 1.37 | 1.11 - 1.70 | <0.001 | 1.21 | 1.07 - 1.37 | <0.001 |
| Obese | 1.66 | 1.43 - 1.92 | <0.001 | 1.46 | 1.27 - 1.68 | <0.001 | 2.30 | 1.85 - 2.86 | <0.001 | 1.70 | 1.32 - 2.20 | <0.001 | 1.60 | 1.37 - 1.86 | <0.001 |
| Psychological distress (K10) |  |  |  |  |  |  |  |  |  |  |  |  |  |  |  |
| Low | Ref. | - | - | Ref. | - | - | Ref. | - | - | Ref. | - | - | Ref. | - | - |
| Moderate | 1.05 | 0.93 - 1.18 | 0.47 | 1.06 | 0.95 - 1.18 | 0.28 | 1.01 | 0.85 - 1.20 | 0.87 | 1.07 | 0.85 - 1.35 | 0.55 | 1.03 | 0.90 - 1.18 | 0.70 |
| High | 0.99 | 0.87 - 1.12 | 0.85 | 0.93 | 0.83 - 1.05 | 0.24 | 0.95 | 0.79 - 1.15 | 0.59 | 0.96 | 0.75 - 1.23 | 0.73 | 1.06 | 0.92 - 1.22 | 0.42 |
| Very high | 1.13 | 0.97 - 1.31 | 0.12 | 1.03 | 0.90 - 1.18 | 0.70 | 1.17 | 0.94 - 1.46 | 0.15 | 1.02 | 0.76 - 1.37 | 0.88 | 1.28 | 1.09 - 1.50 | <0.001 |
| Pregnancy history |  |  |  |  |  |  |  |  |  |  |  |  |  |  |  |
| No | Ref. | - | - | Ref. | - | - | Ref. | - | - | Ref. | - | - | Ref. | - | - |
| Yes | 3.78 | 2.96 - 4.83 | <0.001 | 1.00 | 0.76 - 1.32 | 1.00 | 5.85 | 4.18 - 8.19 | <0.001 | 4.10 | 2.80 - 6.00 | <0.001 | 5.45 | 4.30 - 6.92 | <0.001 |
| History of terminations |  |  |  |  |  |  |  |  |  |  |  |  |  |  |  |
| No | Ref. | - | - | Ref. | - | - | Ref. | - | - | Ref. | - | - | Ref. | - | - |
| Yes | 0.49 | 0.37 - 0.65 | <0.001 | 0.66 | 0.48 - 0.91 | 0.01 | 0.83 | 0.56 - 1.21 | 0.33 | 0.82 | 0.55 - 1.23 | 0.34 | 0.51 | 0.39 - 0.66 | <0.001 |
| History of miscarriage |  |  |  |  |  |  |  |  |  |  |  |  |  |  |  |
| No | Ref. | - | - | Ref. | - | - | Ref. | - | - | Ref. | - | - | Ref. | - | - |
| Yes | 0.64 | 0.48 - 0.85 | <0.001 | 1.22 | 0.88 - 1.68 | 0.24 | 0.51 | 0.34 - 0.76 | <0.001 | 0.97 | 0.64 - 1.47 | 0.88 | 0.92 | 0.71 - 1.20 | 0.54 |
| Menstrual symptoms |  |  |  |  |  |  |  |  |  |  |  |  |  |  |  |
| No | Ref. | - | - | Ref. | - | - | Ref. | - | - | Ref. | - | - | Ref. | - | - |
| Yes | 3.13 | 2.82 - 3.47 | <0.001 | 1.32 | 1.20 - 1.46 | <0.001 | 3.61 | 3.12 - 4.17 | <0.001 | 2.53 | 2.08 - 3.07 | <0.001 | 2.92 | 2.61 - 3.28 | <0.001 |
| History of PCOS |  |  |  |  |  |  |  |  |  |  |  |  |  |  |  |
| No | Ref. | - | - | Ref. | - | - | Ref. | - | - | Ref. | - | - | Ref. | - | - |
| Yes | 0.87 | 0.70 - 1.09 | 0.23 | 0.97 | 0.79 - 1.20 | 0.80 | 0.52 | 0.36 - 0.73 | <0.001 | 0.74 | 0.51 - 1.07 | 0.11 | 1.12 | 0.90 - 1.40 | 0.30 |
| History of endometriosis |  |  |  |  |  |  |  |  |  |  |  |  |  |  |  |
| No | Ref. | - | - | Ref. | - | - | Ref. | - | - | Ref. | - | - | Ref. | - | - |
| Yes | 0.69 | 0.51 - 0.93 | 0.01 | 1.34 | 1.03 - 1.73 | 0.03 | 2.24 | 1.47 - 3.43 | <0.001 | 1.21 | 0.78 - 1.89 | 0.40 | 0.79 | 0.59 - 1.06 | 0.12 |
| Time |  |  |  |  |  |  |  |  |  |  |  |  |  |  |  |
| Survey 1 | Ref. | - | - | Ref. | - | - | Ref. | - | - | Ref. | - | - | Ref. | - | - |
| Survey 3 | 0.99 | 0.88 - 1.10 | 0.81 | 0.94 | 0.85 - 1.04 | 0.23 | 1.81 | 1.54 - 2.12 | <0.001 | 6.47 | 4.87 - 8.61 | <0.001 | 0.76 | 0.67 - 0.87 | <0.001 |
| Survey 5 | 1.55 | 1.32 - 1.81 | <0.001 | 0.87 | 0.75 - 1.01 | 0.06 | 4.28 | 3.38 - 5.41 | <0.001 | 9.61 | 6.81 - 13.57 | <0.001 | 1.04 | 0.87 - 1.24 | 0.67 |

Notes: ESB = English-speaking background; Reference status = Status 4 (pill); A LARC here refers to the use of hormonal long-acting reversible contraception (progestogen-only implant and the progestogen

IUD). Parity was not included in the models due to overlap with pregnancy history.
